# Synthetic materials in craniofacial regenerative medicine: A comprehensive overview

**DOI:** 10.3389/fbioe.2022.987195

**Published:** 2022-11-09

**Authors:** Mohsen Yazdanian, Mostafa Alam, Kamyar Abbasi, Mahdi Rahbar, Amin Farjood, Elahe Tahmasebi, Hamid Tebyaniyan, Reza Ranjbar, Arian Hesam Arefi

**Affiliations:** ^1^ Research Center for Prevention of Oral and Dental Diseases, Baqiyatallah University of Medical Sciences, Tehran, Iran; ^2^ Department of Oral and Maxillofacial Surgery, School of Dentistry, Shahid Beheshti University of Medical Sciences, Tehran, Iran; ^3^ Department of Prosthodontics, School of Dentistry, Shahid Beheshti University of Medical Sciences, Tehran, Iran; ^4^ Department of Restorative Dentistry, School of Dentistry, Ardabil University of Medical Sciences, Ardabil, Iran; ^5^ Orthodontic Department, Dental School, Bushehr University of Medical Sciences, Bushehr, Iran; ^6^ Department of Science and Research, Islimic Azade University, Tehran, Iran; ^7^ Dental Research Center, Zahedan University of Medical Sciences, Zahedan, Iran

**Keywords:** synthetic materials, biocompatible materials, craniofacial regeneration, dental, tissue engineering

## Abstract

The state-of-the-art approach to regenerating different tissues and organs is tissue engineering which includes the three parts of stem cells (SCs), scaffolds, and growth factors. Cellular behaviors such as propagation, differentiation, and assembling the extracellular matrix (ECM) are influenced by the cell’s microenvironment. Imitating the cell’s natural environment, such as scaffolds, is vital to create appropriate tissue. Craniofacial tissue engineering refers to regenerating tissues found in the brain and the face parts such as bone, muscle, and artery. More biocompatible and biodegradable scaffolds are more commensurate with tissue remodeling and more appropriate for cell culture, signaling, and adhesion. Synthetic materials play significant roles and have become more prevalent in medical applications. They have also been used in different forms for producing a microenvironment as ECM for cells. Synthetic scaffolds may be comprised of polymers, bioceramics, or hybrids of natural/synthetic materials. Synthetic scaffolds have produced ECM-like materials that can properly mimic and regulate the tissue microenvironment’s physical, mechanical, chemical, and biological properties, manage adherence of biomolecules and adjust the material’s degradability. The present review article is focused on synthetic materials used in craniofacial tissue engineering in recent decades.

## Introduction

Over the last 30–40 years, synthetic materials have become more prevalent in medical and dental repair applications (Zaszczynska et al., 2020). Generally, biocompatibility, composition, and products released after the decomposition of the scaffolds are highly dependent on the primary materials used for their fabrication (Eltom et al., 2019). Developing biodegradable synthetic scaffolds has omitted the need for additional surgery to remove the guidance conduit (Marti et al., 2013). Hydrogels are porous hydrophilic polymer constructs that efficiently transmit water, gas, and nutrients highly required for cultured cells (Maisani et al., 2017). Hydrogels can also be solidified through a chemical or non-chemical phase transformation; however, they are usually printed in the liquid state (Vega et al., 2017; Francis et al., 2018). The recent substantial progress in synthetic scaffolds has led to producing ECM-like materials that can properly mimic and regulate the tissue microenvironment, manage adherence of biomolecules and adjust the material’s degradability (Oksdath et al., 2018). These controllable properties provide essential advantages for craniofacial tissue engineering (TE). Another reason for the growing application of synthetic materials in dental and oral regeneration is their outstanding reproducibility, immunogenicity, and degradation rates (Marti et al., 2013). TE mainly aims to restore, preserve, or improve the structure and/or function of the damaged tissues due to disease or trauma using scaffolds with or without cells and biochemical factors (Sergey, 2018; Jenkins and Little, 2019). In one meaning, TE occurs at the cross-section of life science and engineering and, importantly, involves the cell-ECM interactions. Involving proteoglycans and fibrous proteins, ECM mainly supports the cells’ structure and morphology, migration or attachment, differentiation, and immune response (Heboyan and Avetisyan, 2019a; Jenkins and Little, 2019; Tanweer et al., 2022; Vardhan et al., 2022; Yadalam et al., 2022). Cells’ homing and proliferation properties highly depend on their microenvironment, and in turn, the biocompatibility of the counterpart artificial ECM. Bioengineered and cell-free scaffolds are two types of artificial ECM. The former is usually supplied with cells, growth factors, cytokines, and/or genetic material before implant (Yaremenko et al., 2018; Soufdoost et al., 2019; Mosaddad et al., 2020; Soufdoost et al., 2020). The three-dimensional (3D) porous biodegradable scaffold is a modern TE technique in clinical dentistry that overcomes the drawbacks of previously introduced cancellous or particulate graft materials. Such scaffolds provide the proper physical space for regenerating the craniofacial tissues, prevent the regeneration site from undesired cells’ invasion, help the endogenous osteogenic cells to reside, grow, and differentiate in the site (Yaremenko et al., 2018; Heboyan and Avetisyan, 2019b; Heboyan, 2019; Heboyan et al., 2019; Abhay et al., 2021; Heboyan et al., 2021a; Avetisyan et al., 2021; Durairaj et al., 2021; Heboyan et al., 2022a; Ahmed et al., 2022; Baskaran et al., 2022; Dam et al., 2022; Gopalakrishnan et al., 2022). The most recent cell-scaffold technique is the personalized scaffold that is fabricated from synthetic polymers such as polycaprolactone (PCL) by 3D bioprinting systems (3DPs) in the precise shape and size. They present valuable features such as thermoplasticity, biocompatibility, biodegradability, and the potential of layer-by-layer processing which have provided a wide application for them in the scaffold fabrication area (Yaremenko et al., 2018). Accordingly, the promising outcomes of TE and clinical regenerative medicine, especially for bone regeneration purposes, have prompted researchers and clinicians to develop such intervention strategies for repairing complicated craniofacial defects despite their associated limitations with synthetic materials. Different synthetic materials and scaffolds have been developed to overcome these limitations. In this review, the recent progressions are discussed in dental and oral regenerative medicine using synthetics materials and scaffolds.

## Craniofacial tissue engineering

The craniomaxillofacial region contains various tissues such as cranial and facial bones, skin, muscles, blood vessels, tendons, ligaments, and nerves (Gaihre et al., 2017; Marya et al., 2022a; Marya et al., 2022b; Ghandhi et al., 2022). As is general in various TE techniques, stem cell (SC), scaffold, and growth factor (Dissanayaka et al., 2015) are the three essential components required for craniofacial TE (Gao et al., 2014). 3D porous bioscaffolds are introduced as appropriate matrixes for this purpose that ideally support attachment, propagation, and differentiation of cells, developing new blood vessels and forming new tissue. In the development of medical technology tailored to each patient’s needs, 3D/4D printing can be combined with artificial intelligence and machine learning (Tatullo et al., 2019; Rokaya et al., 2022). Mesenchymal stem cells (MSCs) are a promising and easily accessible cellular component for craniofacial TE, which, despite their limited self-renewal, they are capable of multi-lineage differentiation. Therefore, different tissues, including bone, cartilage, muscle, fat, nerve, and vessels, can be developed during craniofacial regeneration using MSCs; however, they require a considerable time to expand and undergo aging (Cooper et al., 2019; Soudi et al., 2021; Hussain et al., 2022). In the following, several tissues are present in the craniofacial part of the body, and the studies aimed at regenerating them are implied.

### Muscle

Muscle TE provides attractive models for disease studies and drug screening, regenerative therapies, and meat production (van der Schaft et al., 2013). Various materials have been used as a scaffold for skeletal muscle TE from synthetic polymers or biomimetic materials. The polymers mainly used for such purposes include polypyrrole (PPy), polyglycolide (PGA), polycaprolactone (PCL), and decellularized ECM materials (Grasman et al., 2015; MacQueen et al., 2019; Saberi et al., 2019). At the same time, the biomimetic scaffolds used for the same aim include ECM derivatives such as collagen (COL), fibrin (the most abundant structural protein in skeletal muscle), polysaccharides such as HA, CS, keratin, alginate, fibrous gelatin, and decellularized matrices (Fuoco et al., 2016; Laternser et al., 2018; Nakayama et al., 2019). The specific skeletal muscle from the craniofacial region has been mostly regenerated using PCL-COL nanofibers as a promising scaffold that correctly simulates the skeletal muscle structure and promotes MSCs and myoblasts to align parallelly (Witt et al., 2017). Acellular skeletal muscle ECM has also been reported as a promising scaffold for musculoskeletal TE (Cheng et al., 2014).

Regarding the cells for craniofacial skeletal muscle regeneration, autologous laryngeal muscle repair and reconstruction have been done by primary multi-lineage progenitor cells (Brookes et al., 2018). Also, the gingiva-derived mesenchymal stem cells (GMSCs) can remodel scar/fibrosis and regulate the function of endogenous myoblast progenitor cells to facilitate tongue regeneration (Xu et al., 2017a). Other SCs, including adipose-derived stem cells (ADSCs), induced pluripotent stem cells (iPSCs), and embryonic stem cells (ESCs), have also been used for craniofacial skeletal muscle regeneration (del Carmen Ortuño‐Costela et al., 2019; Kesireddy, 2016; Christ et al., 2015).

### Bone

**FIGURE 1:**
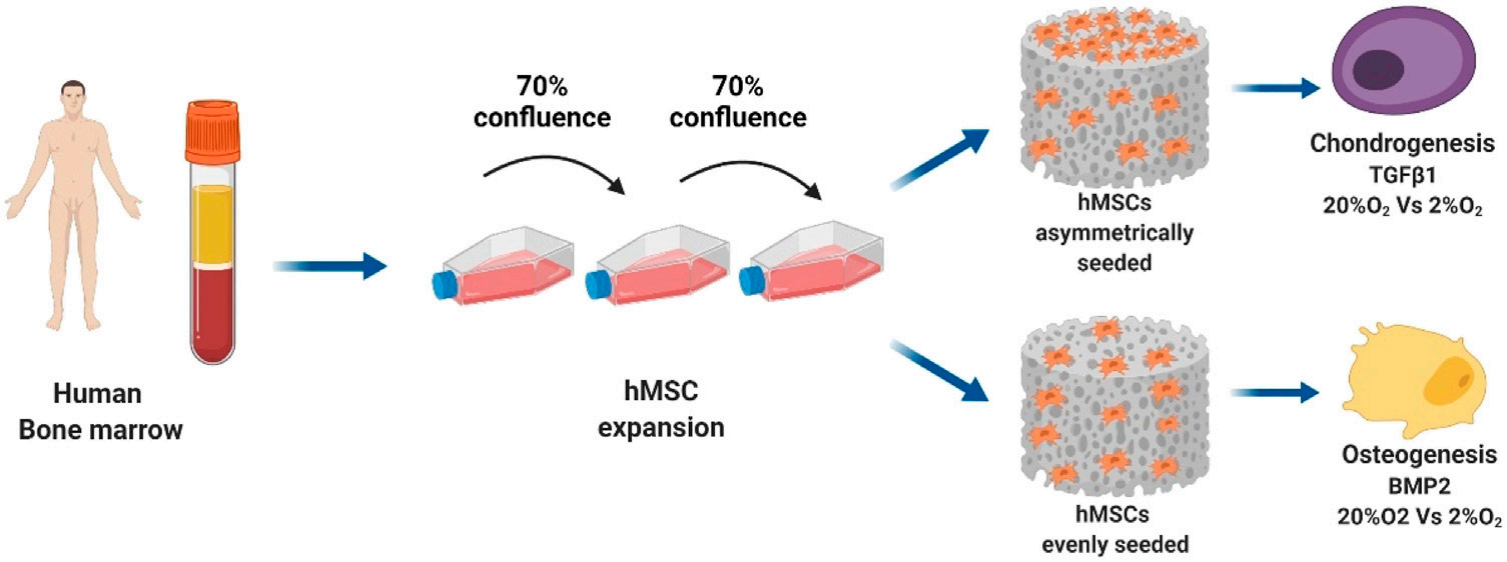
The bone TE paradigm. Scaffold, stem cells, and bioreactor culture systems enable the growth of grafts *in vitro* for bone and cartilage repair applications *in vivo* (Monaco et al., 2021).

The previous limitations and problems with the incorporation of cells, osteoconductivity, osteogenicity, and osteoinductivity in traditional grafts have triggered several pieces of multidisciplinary research on developing new tissue grafts and scaffolds over the last decade. Researchers from various specialties, such as clinicians, engineers, material scientists, chemists, and molecular biologists, have attempted to produce allogenic bone grafts using either mineralized or demineralized bone matrixed GFs, and SCs (Figure 1) (Gali et al., 2018). In addition, structural, mechanical, and spatial stimulation of the signal required for proper bone regeneration is exerted *via* developing natural-based nanocomposites (Gali et al., 2018; Fragogeorgi et al., 2019). Despite the significant limitations of bone TE, such as lack of reproducibility, long-term viability, osseointegration, degree of internal revascularization, and anastomotic potential, ongoing research continues to construct an ideal engineered bone tissue (Mercado-Pagán et al., 2015). In this regard, several groups have utilized the calvarial bone defect in rodent species to assess various types of scaffolds in combination with or absence of cells or GFs (McGovern et al., 2018). The strategies for bone repair are categorized into eight groups based on the trauma extent they cause, including singular synthetic substitution, fibrous/nonfibrous biomimetic substitutes, scaffold-active molecule combination, nano-medicine, SC-based products, 3D cell-printing biomaterials, bioactive composites (can be either porous polymers or inorganic), and finally, nano-scaffolds combined with magnetic field and SCs (Ansari, 2019). Moreover, some ECM cell-binding proteins (e.g., COL I and vitronectin) have been shown to have osteogenic potential (Lopes et al., 2018). The bone TE paradigm comprises an osteoinductive scaffold, an osteogenic cell source, and a bioreactor that provides biophysical stimuli and improves transport. Graft survival and bone regeneration *in vivo* could be enhanced by promoting the formation of functional blood vessels perfused with blood and by stimulating the natural sequence of inflammatory and anti-inflammatory signals that positively interact with the inflammatory response (Ng et al., 2017).

**FIGURE 2:**
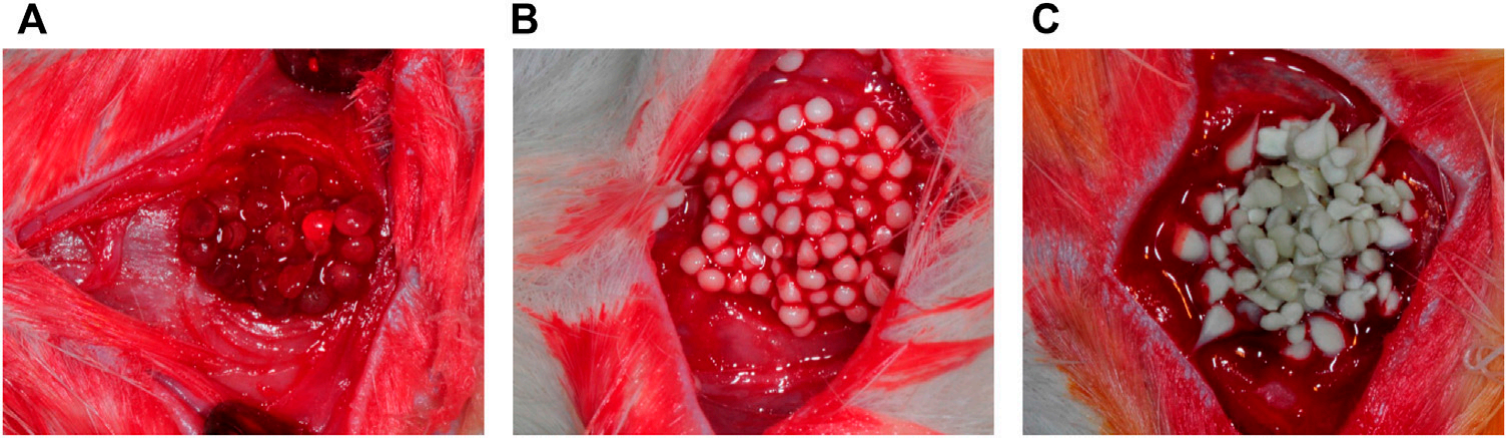
**(A)** Alginate; **(B)** HAp/Alg; and **(C)** Silk Fibroin/Hydroxyapatite particles/Alginate beads were grafted into rat calvarial defects (Jo et al., 2017).

Currently, autografts and allografts are used for craniofacial bone TE, but reconstruction with these standard methods may lead to donor site morbidity and flaws to host tooth aesthetic characteristics (Gaihre et al., 2017). In addition, conventional treatments using bone TE scaffolds face challenges such as high expenses, complicated fabrication, unmanageable degradation, inflammation risk, and immunity rejection that may require debriding by surgery (Fu et al., 2019). Alg/HAp/SF are examples of effective scaffolds for bone TE applications (Figure 2) (Jo et al., 2017).

Biomaterials used in TE should provide certain properties in biological, mechanical, and osteoinductive qualities. Documented interactions between various biomaterials and targeted cell types get involved in the selection scaffold for different clinical cases (Roi et al., 2019). The signaling mechanisms contributing to the coupled angiogenesis and osteogenesis during bone healing involve several cell types, including MSCs, osteoblasts, osteoclasts, endothelial cells, and osteoprogenitor cells (Marrella et al., 2018). In addition, several growth factors (frequently BMPs) are used in TE. Some other examples are FGF-2, VEGF, TGF-β1, -β2, and -β3, PDGF, IGF-1, and SDF-1 (Samorezov and Alsberg, 2015). PTH1-34 is another GF shown to increase the MSCs’ proliferation and differentiation in the primary phases of bone healing (Takahata et al., 2012). BMP-2, BMP-4, BMP-7, and rhPGDFBB are the main GFs used in clinical procedures (Ansari, 2019). Finally, there is a considerable number of methods available for fabricating bone TE scaffolds. Some examples are electrospun nanofibrous scaffolds and rapid prototyping technologies such as SLA, DLP, SLS, and 3DP (Yuan et al., 2017; Udomluck et al., 2020). Bone matrix production shows differences in endochondral and intramembranous grafts suggesting a site-specific bone formation pattern based on the form of cranial bone ossification (Thompson et al., 2017). Osteoconduction and osteoinduction properties of demineralized bone matrix (DBM), especially from cortical bone have made it a proper osteoinductive matrix (Ansari, 2019). HAp crystals and dentin matrix proteins (DMPs) are prerequisites for forming vertebrate bio-minerals (Ravindran and George, 2015). Other supporting factors for bone formation are MSCs combined with α-TCP that help bone TE strategies to be favorably comparable with autogenous bone transfer (Raposo-Amaral et al., 2014). Also, recent studies introduce bisphosphonates as beneficial reagents for bone TE, especially when associated with composite 3D scaffolds (Cattalini et al., 2012).

### Cartilage

Hyaline cartilage is an avascular tissue characterized by copious ECM, primarily composed of COLII and PG, and the chondrocyte, which can retainthe integrated form of ECM (Phull et al., 2016; Vaca-González et al., 2019). Several defects may occur for articular cartilage in terms of depth, size, and the extension of injury that necessitate providing effective medications to repair and restore the defects. The platforms used as a framework for articular TE should have essential qualities such as porosity and inter-connection to conduct cell differentiation and support tissue mechanical properties. Moreover, cell attachment is guaranteed by the surface properties of the materials and scaffolds. Biocompatibility, biodegradability, and non-toxicity of byproducts during the matrix degradation are other necessities for proper contact with the *in situ* native compartments such as COL fibers (Doulabi et al., 2014; Wang et al., 2018a). Chondrocytes are also used in scaffold-based approaches, including porous matrixes that form neo-cartilage within the pores after degrading the scaffold. Generally, two types of scaffolds are used in cartilage TE: microporous scaffolds (hydrogels) and macroporous scaffolds. Hydrogels can contain water as much as articular cartilage making them a standard option for cartilage regeneration *in vitro* and *in situ* (Xiao et al., 2013; Bernhard and Vunjak-Novakovic, 2016; Huang et al., 2016). However, using hydrogels obtained from solubilized dECM is commonly associated with poor mechanical properties and dense ECM that challenges the cartilage decellularization and later cell seeding (Kim et al., 2019a). One of the few methods that have successfully improved the limitations of hydrogels is making pepsin-soluble hydrogels obtained from decellularized cartilage and ligament ECM. Although, the chondrogenesis may decrease in this method compared to the gelatin methacrylate (GelMA) method (Cheng et al., 2014). To compensate for this limitation, several chondrogenic factors such as transforming growth factor-beta (TGF-β), fibroblast growth factors (FGFs), insulin-like growth factor-1 (IGF-1), wingless-type (Wnt) family members, and cartilage-derived matrix proteins (CDMPs) have been used (Grasman et al., 2015; Brookes et al., 2018). Nanofibers, developed with electro-spinning advances, have been broadly connected in cartilage TE (Eftekhari et al., 2020). Bone-like scaffolds and self-organized biphasic cartilage in conjugation with a HAp layer are other materials used for improving articular cartilage injuries and osteochondral defects (Kumai et al., 2019). Additionally, photo-cross-linkable chondroitin sulfate-methacrylate (CSMA) and methacrylated hyaluronic acid (MAHA) have shown the ability to strengthen the mechanical properties of cell-based hybrid-structured scaffolds for cartilage TE *in vitro* and *in vivo* (Kim et al., 2017a).

### Temporomandibular joint disc

Mandibular jaw joints or TMJs, including the mandibular condyle’s cartilage, are mainly composed of fibrous tissue rather than hyaline cartilage at the articular surfaces. They have special features such as embryonic origin deriving from cranial neural crest (CNC) cells, lower COLI content, and COLII-free superficial layers (Suzuki and Iwata, 2016). TMJ may be conflicted with a heterogeneous cluster of diseases such as severe osteoarthritis and rheumatoid arthritis and disability of masticatory functions due to weakening. TMJ disc has a unique structure involving condyle and glenoid fossa that is appropriately fit at their articular surfaces (Wang et al., 2012). Temporomandibular disorders (TMD) affect joints and their associated structures, as in two-third of cases, the TMJ disc is involved (Matuska et al., 2016; Rokaya et al., 2018a). Simple treatments can mainly treat TMDs, but no successful substitution is available for TMJ disks yet (Ângelo et al., 2017). However, regenerative medicine attempts to provide a clinical alternative to TMJ disc resection using complex biochemical and biomechanical *in vitro* approaches (Willard et al., 2012). The significant challenges in this regard are the lack of adequate collagen production and the inability to imitate native mechanical properties. On the contrary, biomaterial approaches have been likely more successful in imitating the native features of TMJ disc, but their viability *in vivo* remains to be determined (Lowe and Almarza, 2017). For example, a unique scaffold has been designed by Van Bellinghen et al., which is suggested to be a perfect approach for TMJ restoration. This complex scaffold is functionalized with dynamic osteochondral molecular gradients such as IGF-1, TGF-1, and bFGF and contains a single SC population that can differentiate toward osteogenesis and chondrogenesis. These characteristics help orchestrate a natural-like 3D arrangement of TMJ tissues (Van Bellinghen et al., 2018). Other methods used for TMJ disk regeneration include matrix-associated chondrocyte transplantation, biologic ECM scaffold obtained from the xenogeneic urinary bladder, acellular scaffold implantation, avascular graft scaffold, and TMJ-derived synovial stem cells (TMJ-SDSCs) (Brown et al., 2012; Xiao, 2014; Bhumiratana et al., 2016; Akar et al., 2018; Undt et al., 2018). Poly glycerol sebacate (PGS) is another potential material to be used as a scaffold for TMJ disc TE (Hagandora et al., 2013). Moreover, Wu et al. could repair the TMJ disc perforation by seeding TMJ synovium-derived MSCs in a fibrin/chitosan scaffold (Wu et al., 2014). Recent studies have also demonstrated the potential of a cell-seeded non-woven mesh made of PGA for application as a scaffold for TMJ disc TE (Mehrotra, 2013). In another report, primary chondrocytes driving from distinctive zones in articular cartilage were seeded in layers in an agarose hydrogel. The coming about neocartilage imitated zonal biochemical and cellular organization (DuRaine et al., 2015). Scaffolds have been used with hyaline cartilage and condylar cartilage to compare their regenerated tissues’ quality and collagen level. While the fibrocartilaginous tissue regenerated by hyaline cartilage-seeded scaffold contained both COLI and COLII, the fibrous tissue regenerated by condylar cartilage-seeded scaffolds contained COLI (Murphy et al., 2013). As a result, TMJ’s condylar nature is supposed to be better regenerated using the condylar cartilage-seeded scaffolds, in contrast to the ankle with a hyaline cartilage nature.

### Salivary glands

Progress in regenerative pharmaceuticals has encouraged studies on improving bioengineered SG TE for transplantation, which might give a selective venue for treating xerostomia (Gao et al., 2014). Non-epithelial stem cells can be differentiated into structurally and functionally effective acinar cells, potentially substituting damaged or lost SG tissues (Xu et al., 2017b).

### Skin

Collagen is the most abundant ECM protein in the human body. The main collagen content of the skin is collagen type I (Guazzo et al., 2018) and III (COLIII), whereas cartilage is mainly comprised of collagen type II (COLII). Therefore, COL-based scaffolds have been widely used for skin tissue regeneration (Law et al., 2017). The epithelial nature of components and fibroblasts are two critical factors in epithelial autografts cultured for skin reconstruction (Mohamed and Xing, 2012). Dermal tissue regeneration is required for severe cases of skin loss such as extent burn wounds. Self-assembling peptide-based hydrogel scaffolds have been used, showing promising potential for accelerating the proliferation of skin cells and wound healing (Chaudhari et al., 2016). Also, the combination of nano-fibrous membranes with fibrin nano-coating and ascorbic acid appears to be particularly helpful for dermal TE (Bacakova et al., 2016). Polyhydroxybutyrate (PHB) combined with organic soluble chitosan (CH), tropoelastin in connection with COLI, COL cross-linked CS, and heparin cross-linked COL with or without growth factors (e.g., FGF-2 or VEGF) are other attempted scaffolds that have shown to be advantageously involved in skin repair (Bi and Jin, 2013). These studies have successfully led to an actual product: MatriDerm® is a natural dermal graft made up of bovine collagen and an elastin hydrolysate. This skin autograft has provided a single-stage procedure for dermal repair in burn wounds (Chua et al., 2016). Other attempted therapies for skin TE are stem cell-based treatments, especially using adipose-derived stem cells (ASCs) (Klar et al., 2017).

### Nerve

The treatment of facial paralysis has dramatically advanced in recent years using tissue-engineered constructs (Langhals et al., 2014). For instance, the high biocompatibility, natural biodegradation kinetics, and adjustable chemical properties of collagen make it a suitable scaffold for neural TE (Boni et al., 2018). Some other materials used as a scaffold for neural regeneration include innovative scaffolds (e.g., piezoelectric scaffolds) (Zaszczynska et al., 2020) and graphene nanocomposites (Karimi et al., 2016). Additionally, different SC types, including ESCs, NSCs, human iPSCs, MSCs, and ASCs, have shown promising results for application in neural TE (Karimi et al., 2016). Cell-based therapy using neural crest precursors of rat bone marrow is another likely successful repair method for peripheral nerve defects (Shi et al., 2019). The ability of dental pulp stem cells (DPSCs) to express neuronal markers, support neural growth, and differentiate into Schwann cells has made them promising candidates for cell-based therapies for peripheral nerve defects (Martens et al., 2014; Luo et al., 2018). It seems that the neural crest origin of DPSCs, similar to the origin of cells forming the peripheral nervous system, enables them to differentiate better into the neural and glial cells (Bhangra et al., 2016).

### Blood vessel

Vascular regeneration is a crucial challenge required to be overcome in large tissue engineering applications because deprived perfusion drives cells into necrosis or ischemia. The problem is more severe in the central parts of the graft due to inadequate support of oxygen and supplements (Avolio et al., 2017). Studies report a greater volume of successful results on vascular TE on small scales. Tubular structures of vessels are generally regenerated through three strategies: sheet rolling, tubular molding, or direct scaffolding (Song et al., 2018). Scaffolds such as silk fibrous, COL, gelatin fibrous, and elastin have been used for small-diameter blood vessel fabrication (Awad et al., 2018). Moreover, a wide range of polymers from PGA-derived materials such as PGA/poly L-lactic acid (PLLA), COL/elastin, CS, PGS, poly glycolide knitted fiber, and L-lactide/ε-caprolactone copolymer have been used as a scaffold for vascular TE. Amniotic fluid has been mentioned to decrease the fabricating time of scaffold-based blood vessels using autologous cells (Nemeno-Guanzon et al., 2012). Decellularized blood vessels are other suggested matrixes for vascular engineering (Simsa et al., 2018). Stem cells used for vascular TE can be originated from either progenitor cells or adult stem cells from diverse sources, including bone marrow, adipose tissue, hair follicle, and umbilical cord (Wang et al., 2017). In addition, relevant studies have indicated the critical role of key growth factors (VEGF, Ang 1, BMP-4) in vascularization by different subtypes of endothelial cells in a concentration-dependent manner (Traore and George, 2017).

## Synthetic scaffolds

Synthetic scaffolds may be comprised of polymers, bioceramics, or hybrids of natural/synthetic materials. The main advantages of polymeric scaffolds are biodegradability and manageable degradation time. Nevertheless, when the pH is lower than 5.6, the produced acidic abused materials stimulate a local inflammatory reaction and dissolve the surrounding apatite, which is the major drawback of these scaffolds. Among the polymeric scaffolds, PLGA, PLCL, and other polyester and their copolymer are widely used (Pangesty et al., 2017; Mirchandani et al., 2021; Shetty et al., 2021; Srimaneepong et al., 2021; Srimaneepong et al., 2022a; Mirza et al., 2022; Mubaraki et al., 2022; Patel et al., 2022; Ramzan et al., 2022; Syed et al., 2022). They can also be produced in different sizes, including nano-scales; however, insufficient ECM-mimicry and possible toxic byproducts after degradation are noticeable limitations of synthetic materials (Marti et al., 2013). Due to the machine’s ability to form sharp corners and clear boundaries, there is a limit on the amount of detail that can be created. In addition to this product’s limited mechanical, residual solvents, and Porogen material properties, there are several disadvantages. There is a more extended time lapse between the process’s beginning and end. It is important to note that they have many disadvantages, including the fact that they are highly susceptible to contamination by bacteria (like endotoxins), lack tunability, react immunologically, degrade at an uncontrollable rate, and do not have a mechanical expectations. In terms of biological aspects, these have several disadvantages in terms of their lack of cellular adhesion sites, so they require chemical modification to enhance the cells’ adhesion to them to improve their performance (Heboyan et al., 2018a; Heboyan et al., 2018b; Heboyan et al., 2020a; Heboyan et al., 2020b; Heboyan et al., 2021b; Reddy et al., 2021; Heboyan et al., 2022b; Heboyan et al., 2022c; Marya et al., 2022c; Heboyan et al., 2022d; Heboyan et al., 2022e; Heboyan et al., 2022f; Kakkad et al., 2022; Karobari et al., 2022). The following describes the most synthetic materials used in craniofacial TE. Moreover, the recent studies of the applied synthetic materials in craniofacial tissue engineering are summarized in Supplementary Table S1.

### Graphene

The field of research seeking novel materials has gained much attention to graphene-based materials. Drug delivery, antimicrobial agents, biocompatibility, and other applications can be achieved with graphene-based nanomaterials. The graphene family is a group of carbon-based nanomaterials with a hexagonal molecular structure containing several derivatives such as Few-Layered Graphene (FLG), ultrathin graphite, graphene oxide (GO), reduced GO, and graphene nano-sheets. In biomedical fields, graphene has many potential uses, which is why it must have good surface adhesion to the surface (Guazzo et al., 2018; Rokaya et al., 2019; Rokaya et al., 2021). The graphene family’s unique mechanical, electrochemical, and physical properties, such as excellent biocompatibility, bioactivity, dispersity, and hydrophilicity, have extended their application in TE (Zhang et al., 2017a). In addition, its capacity to adsorb GFs, drugs, and other biomolecules, because of its superficial functional groups, is much beneficial to TE therapy (Nishida et al., 2016), especially for bone and periodontal tissue regeneration (Kawamoto et al., 2018). Therefore, GO has been combined with other materials, such as poly(vinyl) alcohol (PVA)/CS and methacrylated chondroitin sulfate, to improve the cell growth rate and articular cartilage differentiation (Liao et al., 2015; Zhou et al., 2018). Covalent or non-covalent functionalization is possible for graphene. Nitrogen, carbene, and aryl intermediates can form covalent bonds with graphene. Noncovalent interactions modify graphene, on the other hand. Tissue engineering for bone regeneration and substitution is becoming increasingly popular. As well as bone regeneration, functionalized graphene and its derivatives are also used in tissue engineering and tissue regeneration scaffolds. Functionalized graphene has also been shown to have a high mechanical and therapeutic potential. Supporting specific cells and serving as templates to guide the growth and construction of new tissues are essential features of scaffolds in tissue engineering (Srimaneepong et al., 2022b).

### Calcium phosphate cement

CPC is an outstanding biocompatible, biologically active, mechanically strong, and physicochemically stable synthetic material widely used as a scaffold today (Qin et al., 2016). It has excellent osteoconductive properties and can be ideally resorbed *in vivo* and replaced by regenerated bone (Liu et al., 2013; Osorio et al., 2016). CPC scaffolds are also widely used as composites accompanied by SC types such as MSCs, BMSCs, and human umbilical cord mesenchymal stem cells (hUCMSCs) for regenerating different bone tissues, including cortical osseous autografts (Sun and Yang, 2015; Qiu et al., 2016). BMPs achieve better osteogenic differentiation, non-rigid and strong construct, and optimized mineral synthesis (Thein-Han et al., 2013; Sun and Yang, 2015).

### Polyglycolic acid, polyester poly hydroxyl alkanoate, and poly hydroxybutyrate

PGA is a popular material for scaffolding applications alone or in combination with other materials because of its fast degradation (4–12 months), tensile strength, and proper elasticity (Neto and Ferreira, 2018). PGA has been used in combination with CH for bone TE in extraction sockets (Chang et al., 2014), with collagen for better cellular adhesion (Kinoshita and Maeda, 2013), and PLLA/PCL co-polyesters for cellular-based TE repairing cranial bone defect (Kwon et al., 2015). PHA is an amorphous polymer with biocompatibility, bio‐degradability, and mechanical properties that convey it as a proper candidate for TE applications (Fu et al., 2019). PHB copolymerized with hydroxy valerate (PHBV) has been used for cartilage TE studies and has shown promising results (Xue et al., 2019a).

### Polylactic acid

PLA is an aliphatic thermoplastic polyester with linear polymeric chains, which is susceptible to enzymatic degradation. Its mechanical and thermal properties, biocompatibility, and source sustainability make PLA an affordable and available material for biomedical applications (Sheikh et al., 2015). In an *in vivo* study, the combination of PLA with collagen has been used as a scaffold for seeding with bone marrow-derived MSCs (BMMSCs) and implanted into an animal model. The adequate subchondral bone appearance, cartilage development, and functional repair of deep osteochondral interfacial tissue structure suggested PLA/COL as a potential graft (Kazemnejad et al., 2017). PLLA -A chiral isoform of PLA- has been developed as a dental scaffold model and has become a popular model for dental mechanistic studies and pulp regeneration (Huang, 2009; Demarco et al., 2011; Soares et al., 2018). Also, the injectable nanofibrous form of PLLA microspheres carrying BMP-2 has been shown to improve the regeneration of dentin tissues (Wang et al., 2016a). Wang et al. successfully repaired the mandibular bone defects using nano-HAp/COL/PLA scaffold seeded with alveolar bone marrow SCs (aBMSCs) (Wang et al., 2016b). The potential use of tetracycline-containing fibers for dental implants is beyond their antibacterial properties. However, PLA’s poor toughness and lower elongation at break limit its use for applications requiring plastic deformation at high-stress levels. Researchers have researched to toughen PLLA to overcome these constraints (Rokaya et al., 2018b). PLA has also been seeded with multiple cell types (e.g., Schwann cells and DPSCs) for successfully repairing the nerve defects in an animal model (De Carvalho et al., 2019). PLA is also one of the biomaterials introduced as a potential for TMJ disc substitution, providing a suitable cell adhesion and rearrangement (Ahtiainen et al., 2013).

### Zein

Zein is the main constituent endosperm protein with unique properties such as thermos- resistance, solubility, biocompatibility, porosity, film-forming, and antimicrobial and anti-oxidative effects for scaffold fabrication. Zein scaffold has been used for PDLCs growth for repairing periodontal tissue defects, either alone or with GFs such as *Shuanghuangbu* (a Chinese medicine). *Shuanghuangbu* can enhance the proliferation activity of PDLCs (Yan-Zhi et al., 2010).

### Poly (caprolactone)

**FIGURE 3:**
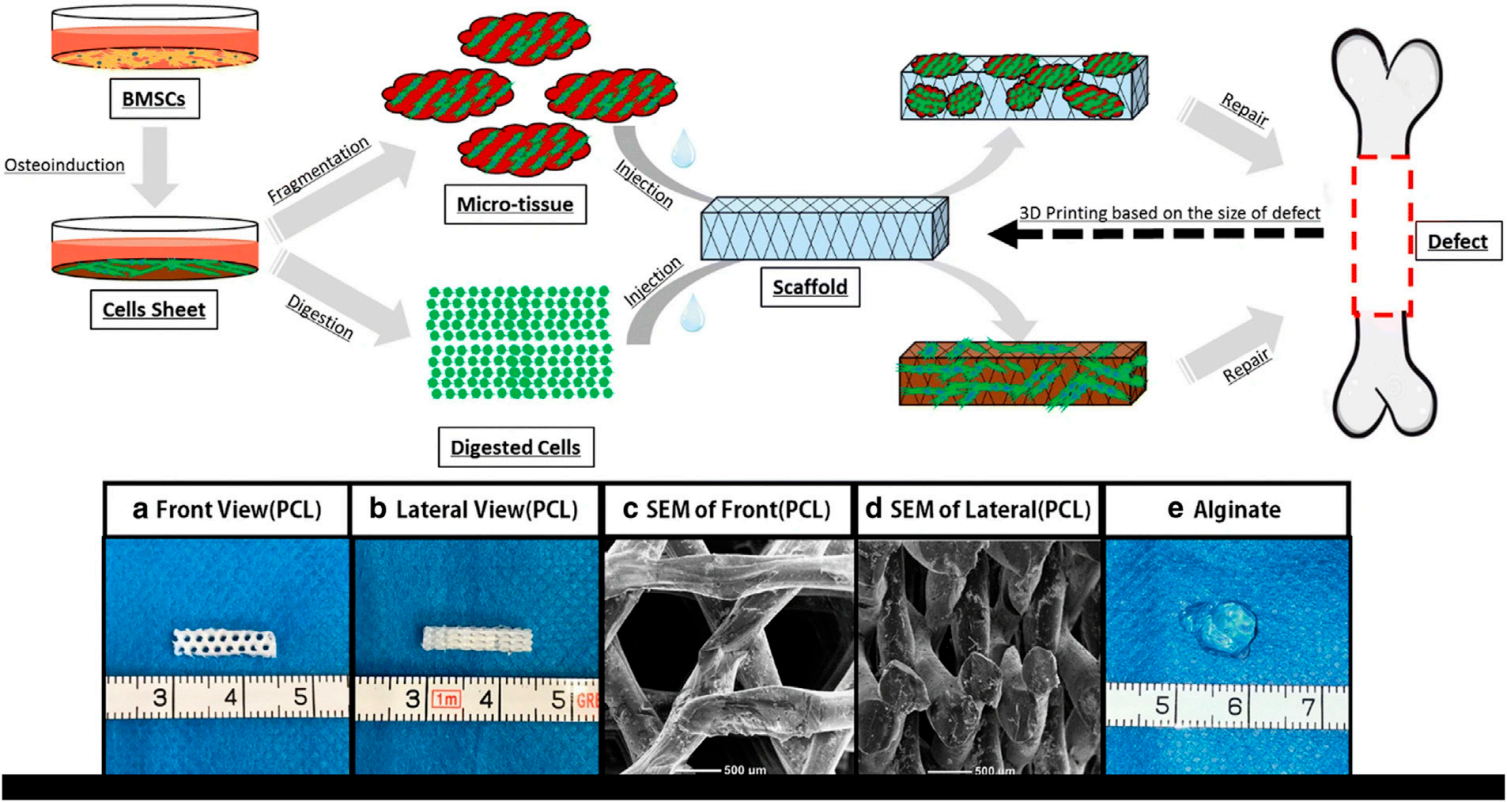
Schematic view of the micro-tissue construction procedures comparison to the digested cells. **(A)** Front view of Polycaprolactone (PCL) scaffold; **(B)** Side view of PCL scaffold; **(C)** A front view of PCL scaffold by electron microscope; **(D)** A side view of PCL scaffold by electron microscope; **(E)** Calcium alginate gel (General view) (Wu et al., 2018).

Slow biodegradability, biocompatibility, and safety approval from Food and Drug Administration (FDA) make PCL an appropriate scaffold for clinical application in medicine and dentistry as well as tissue regeneration (Figure 3) (Jamal et al., 2018; Asa’ad et al., 2016; Wongsupa et al., 2017). Other materials are combined with PCL to improve mechanical, electrical, and biological properties (Flores-Cedillo et al., 2016). Among these additional materials are carbon nanotube (CNT) (Flores-Cedillo et al., 2016), Ibuprofen (Batool et al., 2018), and albumin nanoparticles (Kamath et al., 2014). Albumin-combined PCL has provided promising osteoconductivity and osteoinductivity with a prolonged-release profile ideal for promoting osteogenesis (Kamath et al., 2014). The dose- and spatially-controlled release of antimicrobials and GFs has been investigated by the Ibuprofen-PCL membrane scaffold (Batool et al., 2018). The electrospun membrane forms of PCL have been used to locate the sheet on the dental surface that enhanced adherence between fibrous connective tissue, the regenerated PDL, and bone (Park et al., 2016).

### Poly methylmethacrylate

PMMA has been combined with gelatin and GMPs to carry antibiotics for trauma treatment applications, successfully acting as both porogen and drug carrier. The gelatin and drug solution determine the gelatin swelling percentage and porosity. This construct and its drug release kinetics can be modified and optimized for clinical applications (Shi et al., 2011).

### Polylactide-Co-glycolide

**FIGURE 4:**
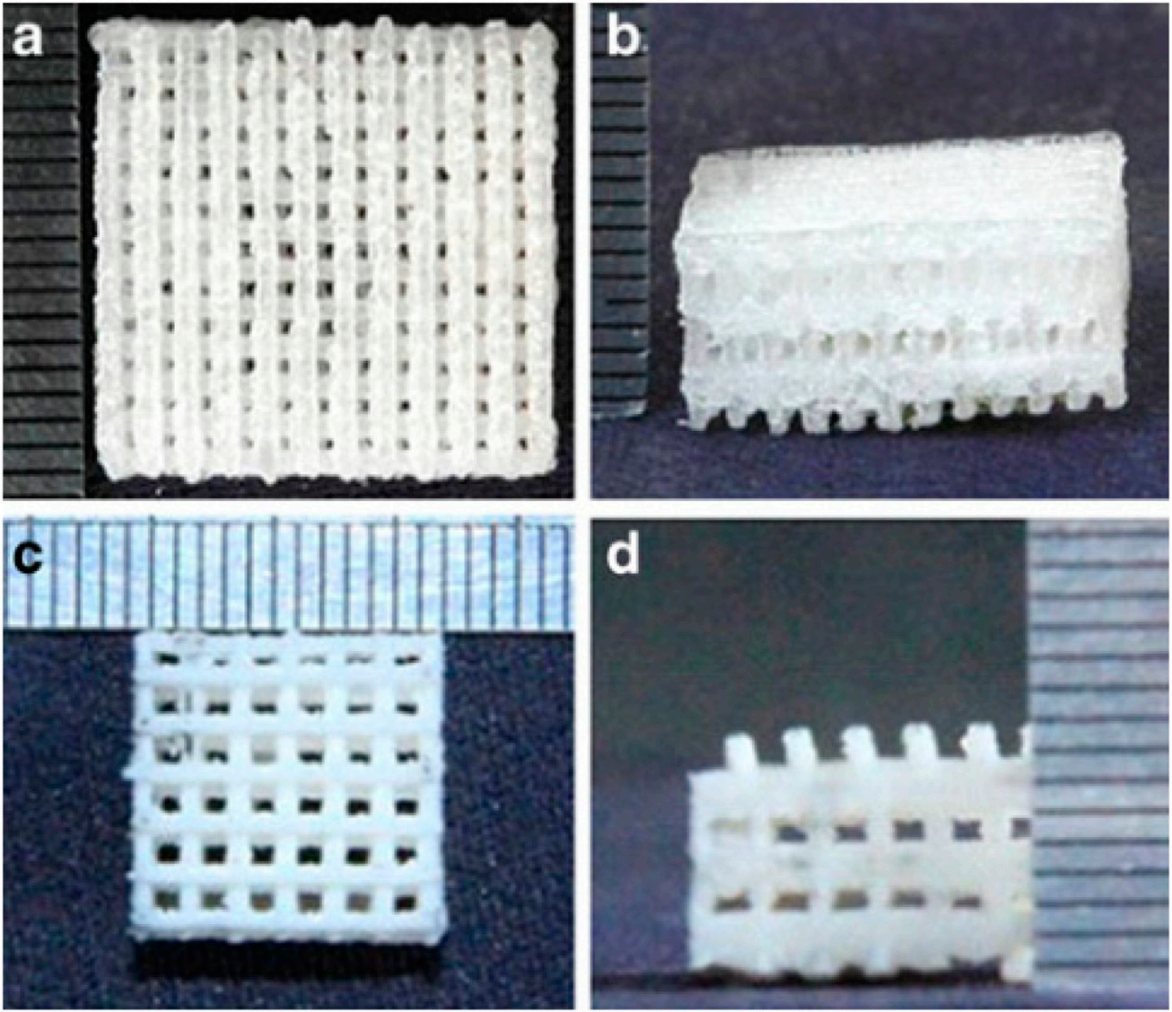
The top and front views of porous poly(lactic-co-glycolic acid) (PLGA) scaffold alone **(A,B)** and in combination with nano- Hydroxyapatite (HA) **(C,D)** (Gentile et al., 2014).

The beneficial mechanical properties, adjustable degradation rates, and high biocompatibility of PLGA (a synthetic aliphatic polyester) make it a suitable scaffold for periodontal therapy (Sood et al., 2012; Campos et al., 2014; Sun et al., 2017). For example, adding HAp can improve the mechanical properties and osteoconductivity of PLGA to meet better bone TE requirements (Figure 4) (Gentile et al., 2014). In another modification try, Cai et al. observed that the ultrasonic treatment of wet electrospun PLGA/PCL scaffolds makes them desirable scaffolds for dental TE (Cai et al., 2017). Also, the combination of Ag nanoparticles with PLGA/CH construct has been employed for periodontal TE. The optimized ratio of PLGA/CS and Ag nanoparticles can achieve high cell mineralization without cytotoxicity (Xue et al., 2019b). PLGA microparticles have also been used for consecutive drug-release at different delivery rates (Santo et al., 2013).

### Poly D, L-lactide, and glycolide

PLG scaffold has been used in an *in vivo* study to regenerate the dental pulp tissue using a heterogeneous population of DPSCs and stem cells from the apical papilla (SCAP). The construct transplanted subcutaneously in the animal model could generate a vascularized pulp-like tissue and dentin-like layer on the canal dentin wall. Additionally, a mineral trioxide aggregate (MTA) cement was observed after a couple of months (Huang, 2011).

### Hydroxyapatite

HAp is one of the vital components of the acellular section of the bone, and then, its synthetic form has been vastly used in bone engineering research (Kumar et al., 2016; Calis et al., 2017). HAp is synthesized in either form of fibrous (by electrospinning) or porous (blended with other biomaterials) (Stratton et al., 2016). The combination of HAp with various polymers, including PLA, PLGA, gelatin, CH, and COL, has been successfully employed for bone formation and arrangement both *in vitro* and *in vivo* (Amini et al., 2012). Since nHAp substantially affects cell proliferation, differentiation, and ECM production considered a bioactive building block of bioengineered dental scaffolds (Lee et al., 2012; van Manen et al., 2014). Also, nHAp and nHAp-coated silk scaffolds have been used for periodontal regeneration (Yang et al., 2013). An avidin-biotin-biotin binding system enhanced the periodontal ligament (PDL) fibroblasts’ adherence to three-dimensionally constructed nHAp scaffolds (Jang et al., 2011). HA surfaces coated with PMP50 were less adhesive and resistant to protein adsorption than surfaces coated with other polymers. PMP films are highly stable and antiadsorptive, making them ideal for dentistry and medicine. Due to its complicated synthesis, MPC is limited in its application because of its high production costs (Rokaya et al., 2018b).

### Poly methoxyethyl acrylate, octacalcium phosphate, and carbon nanotube-based biomaterials

PMEA has been used to reproduce PDL cells showing low adherence to platelets, making it a promising scaffold for TE in blood-rich environments and culturing tissue-derived cells (Kitakami et al., 2014). OCP/COL implantation enhanced bone healing at tooth extraction socket, alveolar cleft model, and mandibular bone defect in a canine model (Kawai et al., 2014). One of the main productive sub-areas of nanomedicine and nanobiotechnology is the biomedical usage of CNT nanomaterials. Outstanding biocompatibility and biophysicochemical properties of CNT-based or -reinforced scaffolds are appropriate for osteoblast distinction and lead bone renewal during bone TE procedures. In addition, the CNTs’ application has been extended as nanoparticles for drug conveyance and cellular transport in tissue engineering, owing to their expansive surface area and great incitement (Pei et al., 2019).

### Gelatin methacrylate

GelMA is the result of combining methacrylate bunches with amine-containing side bunches of gelatins (Rider et al., 2018a). GelMA is frequently used as a scaffold for tissue engineering. Up to now, GelMA’s application has been reported in cartilage TE (Bao et al., 2020), chondrogenic differentiation of BMSCs (Lin et al., 2014), and DSCs and HUVEC differentiation into mineralized dental tissues (Smith et al., 2018a; Smith and Yelick, 2019). Moreover, Smith et al. could produce a mineralized and vascularized tooth bud model by culturing post-natal (embryonic) dental progenitor cell-derived on GelMA hydrogels (Khayat et al., 2017). Similarly, Athirasala et al. could produce pre-vascularized dental pulp constructs on GelMA scaffold using odontoblast-like cells and endothelial colony shaping cells (Athirasala et al., 2017). GelMA-encapsulated DPSC/HUVEC constructs are a promising therapy for pulpal revascularization instead of using devitalized tooth endodontic therapy (Khayat et al., 2017). Cell-encapsulated 3D GelMA hydrogels have also been used with cell sheets (dental epithelial and dental mesenchymal cells). Using this construct could successfully form dental ECM, upregulate the expression of dental cell differentiation markers, and produce biomimetic enamel and pulp organ layers (Monteiro et al., 2016). The GelMA/nHAp microgels have also contributed to periodontal TE *in vitro* and *in vivo*. This construct has promoted new bone formation and facilitated osteogenic differentiation, reproduction, and human PDLSCs viability (Chen et al., 2016a).

### Ceramic scaffolds

Calcium phosphate and hydroxyapatite (HA) are the most widely known and used examples of bioceramic materials recognized as safe substitutes for bone grafts (Nadeem et al., 2013; Murakami et al., 2017). The more flexible bioceramics with progressed mechanical properties are accomplished by adding polymers to bioceramics (Abdulghani and Mitchell, 2019). The osteoconductivity and osteoinductivity of CPCs qualify them for bone tissue repair. HAp, TCP, and their combination as biphasic calcium phosphates (BCPs) and amorphous calcium phosphates (ACPs) are common types of CPCs used in bone TE (Ghassemi et al., 2018).

### Bioactive glass-ceramics

he excellent bioactivity and biosafety of BGC have caused its wide application as scaffolds for bone TE, coatings on metallic implants, and dental filling materials (Mourino et al., 2012; Wang et al., 2013; Zhang et al., 2018a; Kumar et al., 2018; Chocholata et al., 2019). This material is composed of CaO, Na_2_O, SiO_2_, and P_2_O_5_ and can be categorized into resorbable or non-resorbable groups, depending on the relative proportion of its compounds (Siaili et al., 2018). BGC has priority over other ceramic scaffolds, such as HAp and related crystalline calcium phosphates, due to silicon’s ability in osteogenesis management and adjusting mechanical strength and degradation rate (Chen et al., 2012a). For example, bioglass 45S5 has been used for bone formation. The native tissues and bones strongly bond to the carbonate-substituted hydroxyapatite layer on Bioglass’s surface. Over time, bioglass degrades, and new bone is formed (Sriranganathan et al., 2016). Kumar et al. reported that combining bioglass scaffolds and ibuprofen-loaded CTS/nHAp is an improved alternative for TE applications compared to other natural polymer-based scaffolds (Kumar et al., 2019). Moreover, the hybrid of bioactive glass nanoparticles and PCL showed promising results for osteoblast attachment in contrast to the PCL scaffold alone (Lei et al., 2019). Among the coated-bioglass materials with bone regeneration ability, gelatin-coated bioglass scaffolds exhibited the highest cell formation (Zafar et al., 2019). El-Gendy et al. also reported the efficiency of the 45S5 bioglass scaffold for vascularized bone TE (El-Gendy et al., 2015). Additionally, a modified form of 45S5 bioglass-based scaffolds (PHBV-coated) has ideal bone TE characteristics showing increased porosity, proper permeability, and higher pore interconnections (Kashte et al., 2017). As a result of their compositional similarity to bone and tooth structure, bioactive properties, and apparent antimicrobial properties, BAGs have been studied in dentistry as bone substitutes for dentoalveolar and maxillofacial reconstruction, periodontal regeneration, and implants. At present, bone graft surgery uses autologous, allogeneic, xenograft, and synthetic bone grafts. Bone regeneration with autologous bone grafts is considered the gold standard due to its osteogenic properties and absence of immune reactions. There is a limit to how much graft can be harvested because of second bone defects and donor site morbidities. Xenografts, autologous grafts, or allografts cannot achieve the qualities that Janicki and Schmidmaier outlined as ideal graft materials. With the growing demand for BG, the market has begun shifting towards synthetic BGs. Synthetic bone grafts have an advantage over natural bone grafts since they are tailored to meet the needs of bone grafting (Skallevold et al., 2019).

### β-tricalcium phosphate

A key biomaterial for orthopedic and dental applications is β-TCP because of its outstanding biocompatibility and biodegradability (Chanchareonsook et al., 2014; Ke et al., 2015). β-TCP scaffolds are widely studied for personalized craniofacial defect treatment using computer-aided design/computer-aided manufacturing (CAD/CAM) techniques (Wang et al., 2019a). In addition, Henrich et al. have shown a promising application for β-TCP and demineralized bone matrix in treating the bone defect mediated by bone marrow concentrate (BMC) (Henrich et al., 2015). In another study by Su et al., the essential periodontal ligament stem cells (PDLSC) were seeded on β-TCP scaffolds and were able to conduct osteogenesis and adipogenesis (Su et al., 2015). β-TCP has also been used in combination with HAp and COL for producing a viable bone substitute shown to be efficient for preserving the tooth socket after extraction (Ho et al., 2016).

### Pullulan/dextran polysaccharides and calcium silicate cement

A promising substitute with comparable osteogenic properties to HA/TCP ceramics is pullulan/dextran-based hydrogel. These polymers provide fast resorption capacity, a favorable trait for complementary biomaterials used in bone repair applications (Frasca et al., 2017). CaSi-based materials have extensive application in bone TE owing to their osteoconductivity and ability to decrease inflammation markers, especially in primary human DPCs (Chen et al., 2016b; Nagy et al., 2018). These types of cement are widely used for direct pulp capping, root canal sealing, root-end filling, and other endodontic purposes (Tatullo et al., 2019).

### Hydrogel scaffolds

Hydrogels have a wide range of applications, from regenerative endodontics to new tissue formation (Galler et al., 2012). For example, ECM-derived hydrogels are used for bone regeneration and have shown promising results (Pacifici et al., 2018). Single-component self-assembling peptide hydrogels are hydrogels suitable for regenerative periodontal therapy (Koch et al., 2018). Dissanayaka et al. worked on pulp regeneration in an animal-modeled study by utilizing prevascularized Puramatrix^TM^ (a commercially available peptide hydrogel scaffold) and DPSCs (Dissanayaka et al., 2015). Generally, outstanding features of hydrogel-based biomaterials, such as their easy handling, make them desirable for bone regeneration applications, especially in cases of extended defects (Pacifici et al., 2018). In addition, the hydrogel’s capability to transmit biological/chemical factors and entrap progenitor cells such as MSCs or endothelial progenitor cells are highly advantageous for bone TE. Then, some studies aim to enhance their properties by modifying hydrogel structure and stiffness. Hydrogels’ convenient formation and shaping capacity has made them proper scaffolds for short-bone regeneration, such as maxillo-facial bones too (Frasca et al., 2017). Modified hydrogels can change the cells’ affinity for the scaffold or modify their differentiation response (Tan and Hung, 2017). Chitin is a hydrogel scaffold that is extensively employed for upgrading the performance of tissue-engineered constructs (Jayakumar et al., 2011). Another example of modified hydrogels is amelogenin-chitosan, designed and used for the prevention, restoration, and treatment of defective dental enamel (Ruan et al., 2016).

### Nanoparticles

**FIGURE 5:**
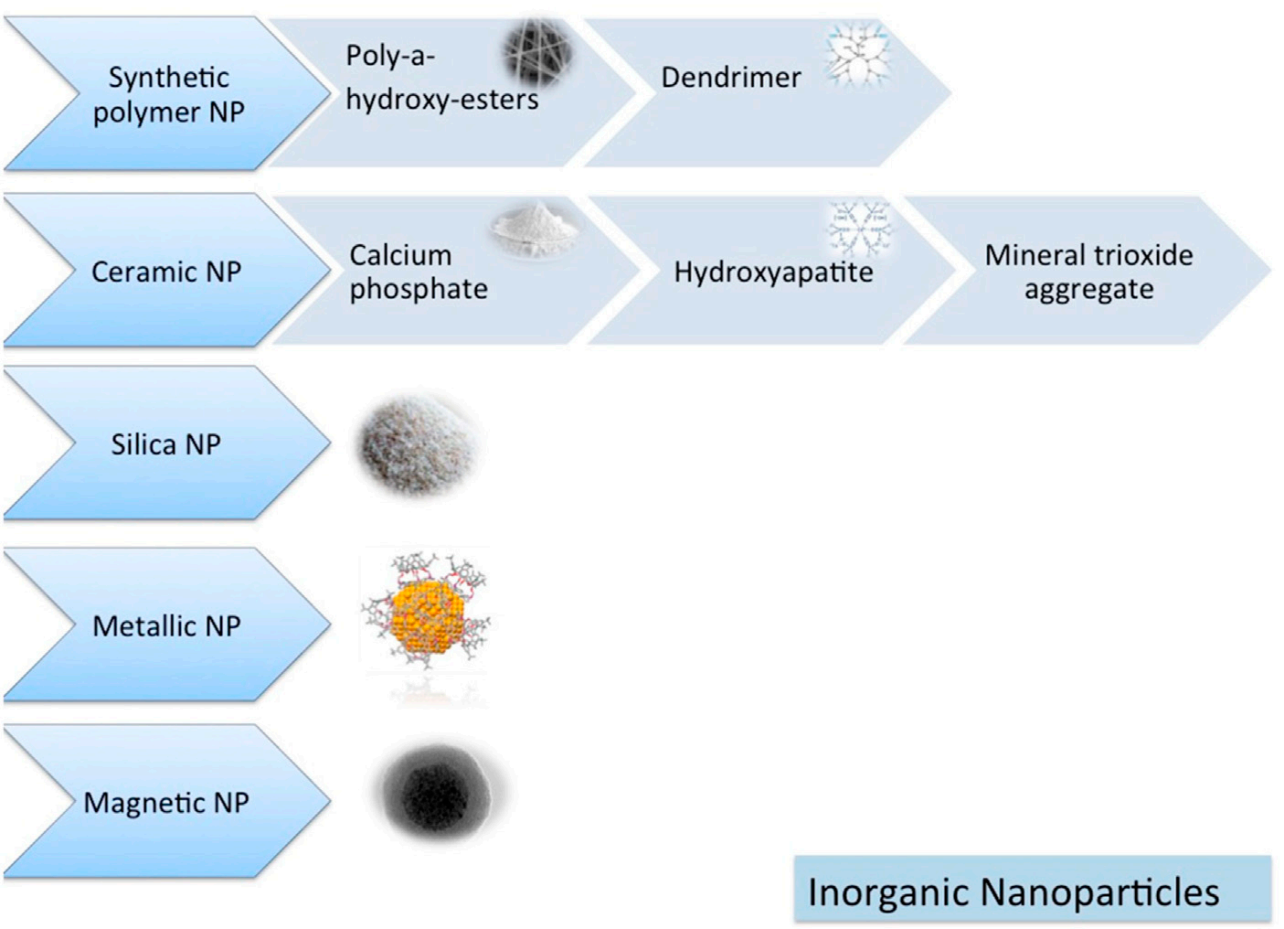
Inorganic nanomaterials (Funda et al., 2020).

**FIGURE 6:**
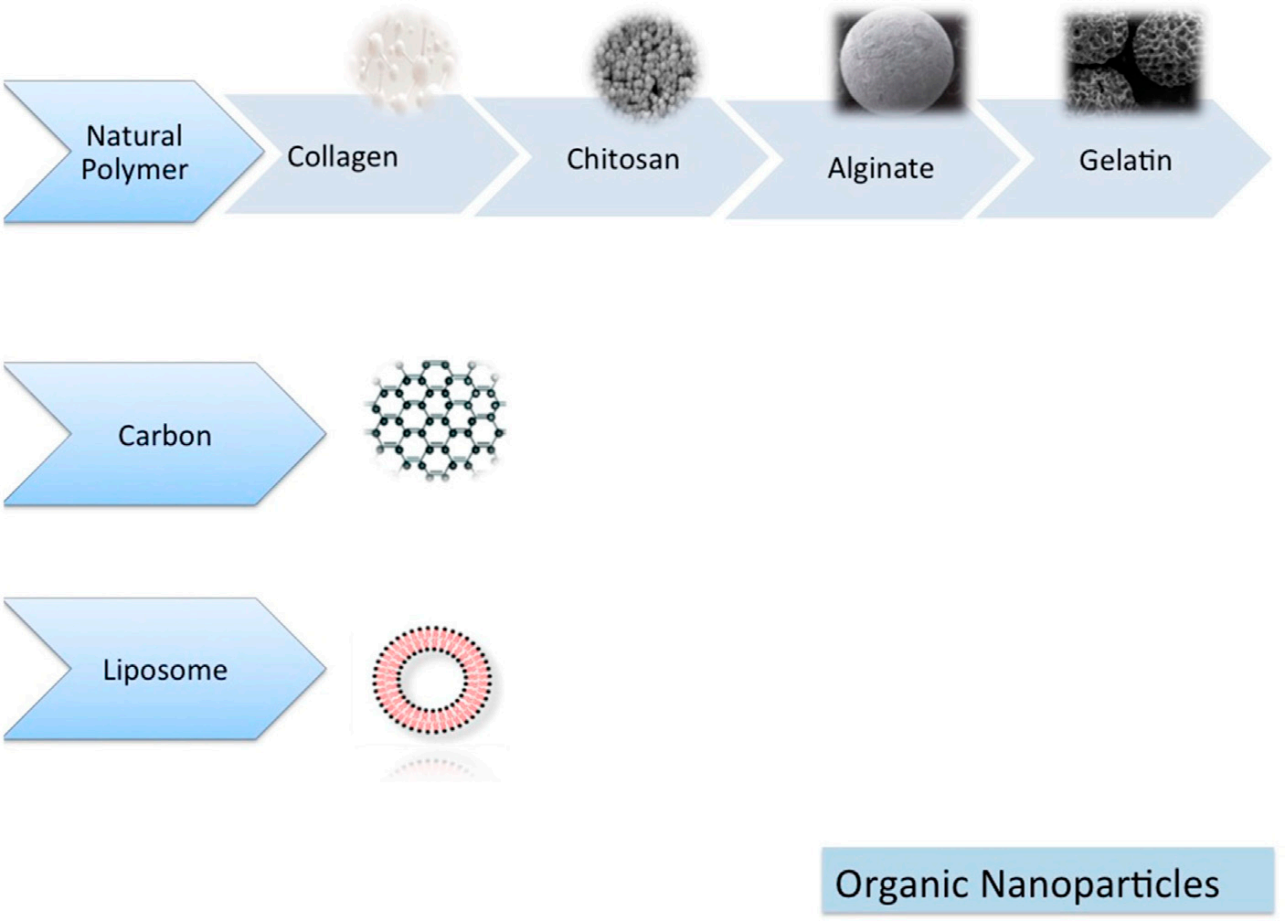
Organic nanomaterials (Funda et al., 2020).

TThe nanoparticle-modified scaffolds have been shown to improve cell growth and tissue engineering, including oral TE, because of their unique physicochemical and biomimetic features (Figures 5, 6) (Funda et al., 2020). The TCP nanoparticle/collagen scaffold is suggested as practical for periodontal TE (Ogawa et al., 2016). CaP nanomaterial scaffolds can mimic the structure of natural bone with increased protein adsorption, higher surface-to-volume ratio, improved wettability, and strengthened mechanical properties compared to older counterparts (Wang et al., 2014). Nano-HAp and granular PRF, in combination with MSC sheets, have been used for the treatment of extensive bone losses. Nanofibrous spongy microspheres seeded with human DPSCs have shown a new perspective for treating dental pulp diseases (Kuang et al., 2016). Carbon nanostructures are other composites shown to be appropriately osteogenic, osteoconductive, and osteoinductive (Perkins and Naderi, 2016). There is an increasing need for MNPs in bone tissue engineering. MNPs, made from metals such as iron and cobalt, can be magnetically manipulated by an external magnetic field. They can enhance their performance using magnetic cores, biocompatible caps, and optional coatings. Various shapes, sizes, and coatings are possible with MNPs, making them highly versatile. Due to their physicochemical properties, they are versatile enough to be applied in various biomedical applications. Since MNPs are intrinsically biocompatible, they have been used in various diagnostic and therapeutic applications. Various methods have been demonstrated in the literature for improving cells, bioactive agents, and scaffolds. In recent years, a way to manipulate cell behavior more accurately and controllably remotely has been explored using magnetic nanoactuation. Incorporating MNPs in scaffold matrices can be accomplished through electrospinning, covalent linking, and freeze-drying. The scaffold-containing MNPs focus on their role in promoting osteogenesis and then on their role in promoting angiogenesis (Dasari et al., 2022).

## Conclusion and future direction

Autografts and allografts are used for craniofacial bone tissue engineering, but reconstruction with these standard approaches may lead to donor site morbidity and flaws to host tooth aesthetic characteristics. The treatments with synthetic materials and scaffolds face challenges, such as high expenses, complicated fabrication, unmanageable degradation, inflammation risk, and immunity rejection. The synthetic materials used in regenerative medicine should provide specific properties regarding biological, mechanical, and osteoinductive qualities. Today, the necessity of developing functional materials and scaffolds for skeletal tissue regeneration is evident regarding the severe deficiency of bone grafts. In this regard, notable scientific progress has been achieved in the past decades, and an assortment of scaffolding materials has been developed for craniofacial TE applications. These materials can provide porosity, biodegradability, cell adhesion, and tissue regeneration. However, they still need to meet some requirements to be clinically approved, which is challenged by the considerable gap between the laboratory and clinic. Moreover, such engineered products’ complexity must be converted to simplicity and feasibility in function and handling. On the other hand, for the best mimicking of the targeted tissue properties, endogenous regenerative capabilities of the host tissues need to be more accounted for. However, considering the complexities of endogenous tissues- especially craniofacial tissues, this is a challenging aim to be achieved. In conclusion, more *in vitro* and *in vivo* studies are suggested to help extend our understanding of the engineered tissue performance in humans and cells’ responding to different parameters.
