# Supplementary material for: Synthetic materials in craniofacial regenerative medicine: A comprehensive overview
Source: Front Bioeng Biotechnol. 2022 Nov 9;10:987195. doi: 10.3389/fbioe.2022.987195 (PMC9681815; doi:10.3389/fbioe.2022.987195)
Supplement: Supplementary file 1 [file Table1.pdf]

Table S1. The summary of the recent synthetic scaffolds in craniofacial tissue engineering.

Type	Stem cells/ Cells	Method	Outcomes	Ref
PLGA coated β-TCP composite	Rat BMSCs	In vitro	Interconnected macroporous scaffolds were fabricated to regenerate bone tissue with enhanced mechanical strength.	(255)
CPC fiber	hUCMSCs	In vitro	An SC-based fibrous scaffold with promising abilities to differentiate and mineralize bones was suggested for repairing orthopedic and craniofacial defects.	(256)
3-D gelatin scaffolds	hDPSCs	In vitro<br>In vivo	The osteo-differentiation potential of hDPSCs was proved. They could form osseous structures on gelatin scaffolds in animal models.	(257)
Polylactic acid scaffolds<br>Bovine collagen scaffolds<br>Calcium phosphate bioceramic	Mature human dental pulp tissue	In vitro	All scaffolds enhanced the mature dental pulp tissue proliferation with a rate depending on their composition (polymer scaffolds showed more optimal proliferation than collagen and CPC).	(258)
Self-assembling peptide nanofibers	DPSCs	In vivo	In vivo implantation of the cell-seeded hydrogel formed a dental pulp-like connective tissue showing firm promises for future regenerative endodontic applications.	(243)
Gel-layered multi-sized β-TCP	Mouse preosteoblast cells (MC3T3-E1)	In vitro	The gelatin-coated scaffold showed more robust mechanical and biological properties.	(259)
Biofunctionalized CPC-alginate-fibrin microbeads	hUCMSCs	In vitro	A novel macroporous construct with a biological function was introduced for bone TE purposes.	(260)
3D nano-fibrous gelatin/silica bioactive glass	DPSCs	In vitro	SCs ideally proliferated on the hybrid scaffold and were suggested to be proper for dentin/pulp TE.	(261)
Polyester copolymer scaffolds	HUVECs, HOB, and CLS	In vitro	The dynamic culture conditions of HOB co-cultured with HUVEC resulted in forming CLS and altering the expression patterns of some osteogenic markers.	(261)
Glass/gelatin composite	hDPSCs	In vitro	The composite scaffold showed proper biocompatibility and promoted hDPSCs osteodifferentiation.	(217)
Premixed calcium phosphate cement (PCPC)	BMSCs	In vivo	The produced complex was suggested for alveolar ridge preservation.	(262)
CPC	Human iPSC-MSCs	In vitro	Cell-seeded CPC was confirmed for bone TE applications.	(159)
CPC	HUVE and HOB	In vitro	The novel constructs enhanced angiogenesis and osteogenesis.	(262)
β-TCP	rhBMP-2 or PRP	In vivo	The biodegradable scaffolds promoted early bone augmentation. However, it could not maintain vertical bone growth, probably due to regenerated bone resorption during scaffold biodegradation.	(263)
CPC	iPSCs	In vitro	The construct was introduced to promote bone regeneration and was suggested for repairing dental, craniofacial, and orthopedic defects.	(264)
Titania-containing phosphate-based glass	Osteoblast-like human osteosarcoma	In vitro	The modified scaffolds claimed to be promising alternatives for bone TE applications.	(265)

3D silanol-functionalized fibers of starch and PCL mixture	Canine ASCs	In vitro	The proposed double-layer scaffold's 3D structure and asymmetric composition were discussed as accounting for selective osteogenesis and recommended for treating periodontal defects.	(266)
3D NF-gelatin/MgP hybrid	DPSCs	In vitro	The nanostructured scaffold could control the metallic ions release conveying it as a promising material for dental TE applications.	(267)
Antibiotic-containing polydioxanone-based polymer	hDPSCs	In vitro	The antibiotic-incorporated scaffolds acted as promising drug carriers introducing them as potential drug delivery systems for endodontic regeneration applications.	(268)
CPC	hESCs	In vivo	CPC was introduced as an efficient approach for craniofacial and orthopedic bone regeneration.	(269)
Arg-Gly-Asp (RGD)-grafted CPC	Human iPSC-MSCs	In vitro	The modified CPC scaffold produced a mineralized regenerated bone for the first time.	(269)
Nanoclay-enriched PCL	hMSCs	In vitro	The enriched PCL showed large bioactive traits ideal for musculoskeletal TE applications.	(270)
β-TCP and HA-coated Zirconia scaffolds	MC3T3-E1 cells	In vitro	The microstructure of zirconia scaffolds had more effects on the osteoblasts' proliferation, differentiation, and activity than the coating materials.	(271)
Modified CPC	Human iPSCs	In vitro	The biofunctionalized cell-seeded CPC showed a high potential for bone tissue regeneration.	(272)
β-TCP	-	In vivo	The doped TCP scaffolds showed great potential for bone TE applications.	(234)
Electrospun chitosan	Osteoblast-like cells (SaOS-2)	In vitro	The genipin-crosslinked CH scaffolds ideally met the degradation time frames for clinical-guided bone regeneration (GBR) applications.	(273)
Synthetic biphasic calcium phosphate (BCP) blocks	-	In vivo	CT scans and CAD/CAM techniques were introduced as helpful methods for producing custom-made scaffolds.	(274)
COL I-coated BCP	Human bone osteosarcoma cell line (MG63)	In vitro	The collagen-combined microporous bone allograft could enhance the cellular function of the bioengineered tissue.	(275)
Pluronic® F127	DPSCs	In vitro	The commercial product- Pluronic F-127- was suggested to be the potential for a cell delivery system for bone TE purposes.	(276)
3D PCL	ADSCs	In vitro	The drug-loaded PCL-HAp scaffolds increased the ADSCs' osteogenesis.	(277)
Mg-CS	hPDLs	In vitro	The modified composite optimally induced odontogenesis and angiogenesis in hPDLs.	(242)
Amorphous TiO_2 NTs on Ti_6Al_4V	Pig periosteal osteoblasts	In vitro	UV and SOW were introduced as effective implant sterilization and disinfection methods.	(278)
Titanium surfaces	BMSCs	In vitro	The surface topography (smooth, micro, and nano) discussed affects the BMSC phenotype and osteoclastogenesis by influencing the local signaling at the implant-cell/bone interface.	(279)
Alginate-CPCs paste	iPSMSCs	In vivo	Cell-seeded scaffolds inoculated with BMP2 could accelerate the scaffold resorption.	(280)

Strontium-substituted glass	-	In vitro	The strontium substituted calcium in the bioactive glasses resulted in retarded apatite-like (octacalcium phosphate) formation, which is the intermediate phase through apatite production.	(227)
Multiwalled carbon nanotubes/PCL composites	hDPSCs	In vitro	The developed scaffold promoted osteogenesis in HDPSCs.	(183)
Triethoxysilypropyl succinic anhydride silane on Ti surfaces	SaOS-2	In vitro	The surface-treated scaffolds biologically sealed the implant against bacteria and provided an effective anchoring platform with improved osteoblastogenesis and antibacterial properties.	(281)
Sr and Ag-loaded nanotubular structures	MC3T3-E1 cells	In vivo	Sr/Ag-loaded nanotubules showed potential for clinical applications.	(282)
PGA fibers	DPSCs	In vitro	The cell-seeded scaffolds provided a potential platform for tendon-like TE.	(283)
nano-β-TCP/collagen	FGF-2	In vivo	The coated and bioactivated TCP NP scaffolds revealed the potential for periodontal TE applications.	(250)
Bovine serum albumin/PLGA/ hexafluoro propanol composite	MSCs	In vitro	The composite scaffolds provided an excellent platform for MSC adherence and proliferation with no toxicity. Neurogenesis-associated gene expression was upregulated in all scaffold groups.	(284)
CPCs	Minipig BMSCs	In vivo	The CPC scaffold was suitable for pBMSCs adherence, proliferation, and differentiation into osteogenic cells.	(162)
Graphene oxide	MC3T3-E1 cells	In vitro	The GO scaffold was biocompatible and had highly osteogenic potential.	(153)
β-TCP	ASCs	In vivo	The regenerated bone tissue effectively treated the peri-implantitis promising for developing stable and masticatory dental implants.	(285)
Gutta-percha nanocomposites	DPSC	In vitro	The nanocomposites were strongly suggested for pulp TE regenerative therapies.	(283)
Alginate-fibrin hydrogel fibers and CPC	hiPSCs<br>hESCs,<br>hUCMSCs	In vitro	The injectable CPC hydrogel fibrous construct encapsulating all three types of cells showed a high potential for enhanced bone regeneration for dental, craniofacial, and orthopedic applications.	(285)
Injectable cell-containing CPC and alginate-fibrin hydrogel	Human iPSC-MSCs<br>DPSCs<br>BMSCs	In vitro	The novel injectable CPC with cell-encapsulating hydrogel fibers was suggested to enhance bone regeneration in dental, craniofacial, and orthopedic applications.	(285)
Poly(LLA-co-CL)	BMSC, BMP2	In vitro<br>In vivo	The recombinant cell-seeded scaffolds showed enhanced osteogenesis potential for bone TE in vivo.	(286)
3D-printed PCL/TCP composite	ADSCs and BMSCs	In vivo	Cell-seeded 3D scaffolds enhanced osteogenesis in animal models with maxillary bone defects.	(287)
Microtubular PMMA	Dental pulp cells	In vitro	The microtubular structured scaffolds were suggested to be useful for clinical applications.	(288)
Titanium-reinforced collagen	rhBMP-2	In vivo	No significant difference was observed in the horizontal bone-gaining ratio.	(289)
β-TCP/HA with or without EMD	-	In vivo	The new scaffold provided an adequate bone regeneration level in the maxillary sinus floor, allowing successful implant installation.	(290)

3D additive-manufactured PCL/FeHAp nanocomposite	hMSCs	In vitro	The magnetic field was reported to be a promising technique associated with applying cell-seeded scaffold for bone TE.	(291)
HAp/PLGA	Autologous MSCs	In vitro	MSC-seeded PLGA composite scaffolds showed enhanced bone regeneration/mineralization and scaffold absorption compared to cell-free counterparts.	(292)
nHAp on PO_2-modified PLGA membranes	Osteoblasts	In vivo	The PO_2-modified and HA-incorporated PLGA membranes promoted enhanced osteogenesis and bone mineralization compared to the single PLGA scaffolds.	(293)
Synthetic BCP	-	In vivo	BCP ideally augmented the maxillary sinus floor and promoted implants' osseointegration.	(294)
BCP, PCL, PLGA, and β-TCP	-	In vivo	Composite bone grafts were introduced as a promising alternative to synthetic bone grafts for clinical applications.	(295)
HAp/β-TCP modified with argon glow discharge plasma (Ar-GDP)	MG-63 cell	In vitro	GDP treatment could effectively disinfect the scaffold's surface and enhance osteogenesis.	(296)
Cell-free biomimetic NF	-	In vitro	Daily pulse delivery promoted extensive bone defect repair with minimal side effects in animal models regenerating new-formed bone of higher quality.	(297)
3-D COLL scaffolds coated with β-TCP NPs	Osteoblasts	In vitro	The bone regeneration ability of NP-coated scaffolds was remarkable.	(216)
P(3HHx-3HO-3HD-3HDD)	Myoblast cell line (C2C12)	In vitro	The novel polymer scaffold promoted increased cell proliferation suggesting as suitable for soft TE.	(298)
Bioactive glass and silk fibroin	-	In vitro	The biphasic scaffold revealed a high potential for repairing the osteochondral tissue in the clinic.	(299)
PCL-BCP	hDPSCs	In vitro	The composite scaffolds enhanced cell viability, adherence, and anchoring and increased alkaline phosphatase activity, osteocalcin production, and mineralization.	(182)
3D-printed medical-grade PCL	Human osteoblasts	In vivo<br>In vitro	Functionalized cell-encapsulated hydrogels formed mineralized tissue acting as acceptable scaffolds for bone TE but showed no significant difference to hydrogels containing BMP-7 alone.	(300)
Nanopeptide hydrogel	Porcine DPCs	In vivo	The developed regenerative microenvironment did not achieve desirable pulp regeneration.	(301)
PLCL	hMSCs	In vitro	An effective technique was introduced for fast cellularization and vascularization.	(126)
CPC	Human iPSC-MSCs and HUVECs	In vivo	HUVECs co-cultured with hiPSC-MSCs substantially promoted bone regeneration.	(302)
Non-woven PCL fabric with apatite surface	-	In vitro	A potential scaffold was suggested for bone TE that provided ideal cell adhesion and penetrability.	(303)
COLL/HAp/β-TCP, jellyfish collagen matrix, and collagen powder	PDLSC	In vivo	CaP-coated implants associated with GBR procedures could enhance peripheral bone growth.	(304)

3D-printed HAp scaffolds	hDPSCs	In vivo	Cell-seeded scaffolds combined with peptide hydrogels promoted osteogenesis and odontogenesis in hDPSCs, angiogenesis, pulp regeneration, and osteodentin deposition in animal models.	(305)
Synthetic apatite (CAP-HA)	-	In vivo	The simulated body fluid could produce a bone-like HAp structure note-worthy for application in bone TE studies and therapies.	(196)
β-TCP scaffolds coated with collagen and PDLLA	-	In vitro	The coating method of bioabsorbable polymers was introduced as a practical approach for increasing the mechanical properties of the scaffold, and collagen helped enhance cell migration.	(306)
Nano-BG, nHAp, and forsterite	-	In vivo	The added nano-groups to the scaffold significantly increased bone regeneration.	(307)
Fe_3O_4 MNP coated with nanoscale graphene oxide	DPSCs	In vitro	The cell-sheet TE approach demonstrated its potential for future regenerative therapies.	(152)
BCP and CMC or HA polysaccharide	-	In vitro<br>In vivo	The composite scaffold showed great potential for bone augmentation applications.	(308)
Electroactive PLLA incorporated with ferroelectric ceramic BTO NPs	BMSCs	In vitro	The importance of several physical properties of scaffolds for optimized mimicking of the osteogenic microenvironment was emphasized.	(309)
Nanocomposite poly-L/D-lactide and HAp bioceramics enriched with sodium alginate	-	In vivo	The used biomaterial and method results were validated for osteochondral repair in the animal model.	(309)
PLA coated with CH/CaSi	hMSCs	In vitro	The electroactive composite showed to be biodegradable and suitable for bone TE applications.	(310)
Collagenated biphasic calcium phosphate	-	In vivo	The synthetic bone substitute used in this study was successfully osseointegrated and was suggested to be useful for sinus augmentation.	(311)
Calcium sulfate ($CaSO_4$)	MSCs	In vitro	Calcium sulfate scaffolds were introduced as a promising matrix for bone TE applications.	(312)
Photo-initiating gelation thiolene hydrogel	An osteosarcoma cell line (G292)	In vitro	This study's established material and methods were suggested as a promising approach for designing orthogonal contexts for regenerative dentistry.	(313)
Osteon growth induction (OGI) titanium surface	MSCs	In vitro	The OGI titanium surfaces increased the expression of markers associated with angiogenesis in MSC and induced proliferation and osteogenesis in the cells.	(314)
Reduced (rGO)-coated BCP	-	In vivo	The BCP coated with rGO effectively conducted osteogenesis and composite concentration.	(315)
MTA	Murine mesenchymal progenitor cells	In vivo<br>In vitro	The injured PDL and alveolar bone were regenerated, and progenitor cells differentiated into osteoblasts in the animal model.	(316)

nHAp, MTA, and calcium-enriched mixture	hDPSCs	In vitro	nHAp showed a less proliferative and more cytotoxic effect on DPSCs than MTA and calcium-enriched mixture biomaterials.	(317)
Bilayer PLGA/nHAp	A murine fibroblastic cell line (L929)	In vitro	The bilayer membrane effectively conducted preferential bone tissue regeneration and showed a high potential for therapeutic approaches.	(318)
β-TCP granules	BMMNCs	In vivo	The graft was safe and effectively applicable for alveolar bone regeneration.	(319)
HAp/TCP and PRP	hADSCs	In vivo<br>In vitro	The scaffold combined with SCs appeared useful for bone TE therapeutic purposes.	(320)
3D-printed PCL and 3D-printed PCL/β-TCP composite	Fibroblasts and preosteoblasts	In vivo	The 3D-printed TCP combined with PCL provided biocompatibility and bone regeneration more effectively than PCL alone and conventional collagen membranes.	(321)
PLA/PGA membrane	HUMSCs	In vivo	Cell-seeded membranes provided root coverage higher than non-seeded membranes.	(322)
3D PCL	-	In vivo	The 3D-printed PCL successfully promoted alveolar bone regeneration.	(323)
PLGA/HAp coated with Asp-Gly-Glu-Ala (DGEA)-incorporated collagen	BMSCs	In vivo	The DGEA/collagen-coated scaffold showed improved properties providing potential for bone regeneration strategies.	(324)
Composite of PHB, chitosan, and nano-bioglass (nBG)	Human deciduous DPSCs	In vitro	The composite was a suitable biocompatible scaffold for dentine TE, especially in combination with growth factors.	(325)
3D printed PCL/TCP	ADSCs and BMSCs	In vitro	The composite scaffold induced osteogenesis in MSCs, showing potential for bone TE.	(323)
Gold nanoparticles (GNP)-CPC	hDPSCs	In vitro	GNPs were introduced as potential bioactive additives for increasing bone regeneration by modifying CPC's nanotopography.	(326)
3D-PLA	hGMSCs, hGMSCs-derived CM	In vivo	The scaffold provided proper context, especially for hGMSCs-derived CM for osteogenesis.	(327)
Synthetic BCP with a HAp/β-TCP	-	In vivo	The higher TCP/BCP content showed more power in inducing osteogenesis for maxillary sinus floor augmentation and positively contributed to bone regeneration.	(328)
GelMA	Odontoblast-like cells (OD21)	In vitro	The hydrogel photopolymerization method used in this study was proposed as practical and essential progress in chair-side procedures for regenerative dentistry.	(329)
CPC containing chitosan and metformin	Human-iPS-derived mesenchymal stem cells and DPCs	In vitro	The CPC-CH-metformin composite was suggested as a promising scaffold for dentin TE and regeneration applications.	(330)
Zirconium oxide (ZrO_2) coated with HAp and impregnated by PRP/heparan sulfate	-	In vivo	In short-term surveys, HA/ZrO_2 showed more osteogenesis ability than PRP alone, while no synergic effects were observed in the long term.	(331)
Laponite®-pNIPAM-co-DMAc hydrogel loaded with nHAp	MSCs	In vivo	The crosslinked hydrogel was biocompatible and site-targeted by promoting cell migration and integration, suggesting potential for safe and efficient bone regenerative and repair.	(332)

Gelatin sponges modified by epigallocatechin gallate (EGCG)	Osteoblastic cells	In vivo<br>In vitro	The bone formation capacity of EGCG was boosted by vacuum heating, providing a suitable scaffold that preserves EGCG pharmacological properties.	(333)
HAp, PRP, BCP, and CH-g-poly(N-isopropyl acrylamide) hydrogel	Rabbit ASCs	In vitro	An injectable thermo-gelling hydrogel was developed and proved to promote osteogenesis efficiently.	(334)
Two calcium silicate-based types of cement (Biodentine and Bioroot)	DPSCs	In vitro	Both biomaterials were represented as bioactive and biocompatible scaffolds that strongly supported SC proliferation, migration, and adhesion and mineralization of the regenerated dentin-pulp tissue.	(335)
Biphasic calcium loaded with PLGA-PEG NPs	BMSCs	In vivo	The synthetically produced nanomaterials acted as an appropriate alternative to autologous bone grafts that helped adjust simvastatin's clinical dosage for bone repair purposes.	(336)
vhEGCG-GS	-	In vivo	The bone formation capacity and the quality of newly formed bone could be determined by altering the composition of vhEGCG-GSs.	(333)
Electrospun PCL	Adult GMSCs	In vitro	The routinely discarded, diseased gingival tissue was introduced as a source for obtaining adult MSCs with practical 'stemness' and osteogenic capacity.	(337)
CH/Gel/glycerol phosphate hydrogel	iPSCs	In vivo	The combined scaffold properly conducted stem cells to the bone through the blood and minimized inflammation, suggesting a potential alternative material for PDL regeneration.	(338)
PCL/Gel/CH/β-TCP electrospun composite	MG63	In vitro	The β-TCP embedded composite scaffold was recommended for GBR applications.	(339)
Osteon II, Tigran PTG, and zirconia grafts	-	In vivo	Zirconia was suggested as a promising synthetic bone graft material for bone regeneration applications.	(340)
FDPC incorporated in a thermo-sensitive CH/β-GP hydrogel	PDLSCs	In vitro	Loading composite hydrogel with FDPC increased scaffold injectability and loaded-cell viability. It also provided a sustained GF-release ideal for periodontium TE.	(341)
Gel and nHAp within porous titanium alloy	MC3T3-E1 cells	In vivo	The modified porous titanium structure provided appropriate conditions for cell growth and a suitable carrier for bioactive factors.	(324)
3D-printed β-TCP with COL ECM	DPCs	In vitro	The hybrid constructs were suggested as a new scaffold for osteoblastogenesis applicable in craniomaxillofacial bone TE.	(342)
Polyethylene glycol/COL/ fibrin	DPSC	In vivo<br>In vitro	Natural materials, especially fibrin, proved superior to synthetic scaffolds regarding cell viability and dental pulp-like tissue formation.	(343)
Calcarea phosphorica doped nBGC	Mouse MSCs	In vitro	Both scaffolds were non-toxic to animal MSCs at lower concentrations. Their bone formation capacity in vitro was better than in vivo.	(220)
PCL	SCAP	In vitro	PCL scaffolds provide a suitable platform for cell adherence, proliferation, and differentiation with no additive required, which is promising for dental TE purposes.	(180)
β-TCP and BCP	ASCs	In vivo	The scaffold provided an efficient platform for stem cell and pro-angiogenesis processes.	(344)

Modified β-TCP	MC3T3-E1 cells	In vitro<br>In vivo	TCP scaffold loaded with 5 mM N-acetyl-L-cysteine provided the best pre-osteoblastic cell viability.	(345)
3D bioprinted alginate hydrogels	SCAP	In vitro	The new bioink was cytocompatible, odontogenic, and capable of fabricating complex 3D microarchitectures for regenerative dentistry applications.	(346)
3D gelatin nanofibers crosslinked with other materials	DPSC	In vitro	The nanofibrous gelatin generated natural-like tubular dentin microstructures and was suggested as a powerful approach for producing functional tissues and studying the interaction of cells and material.	(173)
Poly(LLA-co-CL)	BMSC	In vivo<br>In vitro	The scaffold conducted angiogenesis in cells in the presence of only vascular endothelial GF A but also needed BMP2 to induce osteogenesis.	(347)
CPC	Human umbilical vein endothelial cells/MSCs	In vivo	The co-cultured composites improved angiogenesis and were discussed as having potential for craniofacial/orthopedic bone repair.	(348)
Porcine bone/COL composite	Osteoblast-like cell	In vivo<br>In vitro	The porcine collagen graft was argued to be a promising bone substitute in clinics.	(349)
Augmented bone graft	-	In vivo<br>In vitro	The compression-resistant bone grafts were introduced to be useful for lateral ridge augmentation with no protective mesh required.	(350)
Yttria-tetragonal Zirconia polycrystal	hADSCs	In vitro	The used polycrystal biomaterials were biocompatible and proposed to be implantable in vivo.	(351)
Strontium-releasing fluorapatite glass-ceramic	-	In vivo	The strontium-doped scaffolds could form new bone containing a high amount of fluorine regardless of the crystallization state or composition of the scaffold.	(352)
3D-printed bioactive glass block/nano-CH composites	BMSCs	In vivo	The used composites were properly osteoconductive and could produce new alveolar bone tissue. The osteogenic role of NELL1 was also shown.	(353)
β-TCP and calcium sulfate	-	In vivo	The composite grafts were bioactive and acted as biomimetic alloplastic substitutes for repairing cranial bone defects.	(354)
β-TCP and CS	-	In vivo	The synthetic material used for preserving the alveolar ridge showed stable and adequate biological, functional, and esthetic properties within two years of follow-up.	(355)
β-TCP granules coated with PLGA	-	In vivo	The biomaterial was used as in situ hardening bone substitute in the extracted teeth sockets and shown to be biocompatible and resorbable.	(354)
Graphene-coated Ti_6Al_4V titanium alloy	hMSCs	In vivo	Graphene enhanced the surface bioactivity of Ti-based nanomaterials, which is expected to increase osteogenesis and osseointegration in vivo.	(356)
Functionalized-HAp-coated polyethylene	hBMSCs	In vivo<br>In vitro	Forming bone within the pores of the scaffold was hypothesized to involve the enhanced osteointegration process.	(357)
Bioactive glass/nHAp-reinforced electrospun PCL composite	-	In vitro	The designed membranes were proved to be functional as GTR membranes and promising for developing a clinically applicable membrane.	(358)
Co-polyester-poly(butylene succinate-co-glycolate	MSCs	In vitro	The novel nanofibers were suggested as potential GBR membranes for bone TE use.	(359)

OCP/COL composite	-	In vivo	These results suggest that OCP/Col with the single local administration of parathyroid hormone enhances bone regeneration in a rodent calvarial critical-sized bone defect.	(360)
Octacalcium phosphate with or without gelatin	-	In vivo	The octacalcium phosphate composed of gelatin showed promising results in healing the defects of calvarial bone.	(361)
Triacrylate polymers	DPSCs	In vivo	A polymer microarray method was developed to rapidly identify polymers that can support adhering, increasing, and differentiating cells. These biomaterials were conveyed to be directly applicable at the dentin-pulp interface and suggested for constructing medical devices.	(362)
Chondroitin sulfate (241) gelatin hydrogel	-	In vitro	The CS-gelatin hydrogel showed promising potency for drug delivery and TE applications because of its similarity to natural ECM components such as COL and CH in cartilage.	(363)
GMPs and PLGA microparticles within a CPC	-	In vitro	The macropores generated by embedding GMPs and PLGA in the CPC scaffold reduced the acidity of the post-degradation solution.	(364)
Electrospun PCL and bioactive glass particles	MG63	In vitro	These preliminary results suggested that these new membranes can be a strong candidate for small bone injury treatment by the GTR technique.	(365)
An experimental bone substitute (EBS)	-	In vivo	The EBS made from the inner layer of *Crassostrea rhizophora* was biocompatible and induced a rapid bone formation process in the rat models.	(366)
Fe_3O_4 NPs-loaded PLLA	-	In vivo	The Fe_3O_4/PLLA nanofibers were considered valuable biomaterials for future bone TE applications.	(367)
PCL-based Pus	-	In vitro	The used membranes showed comparably potential properties for GBR purposes.	(368)
Cross-linkable Gel and functionalized GNPs hydrogel	hASCs	In vitro	The designed hydrogels were recommended for various TE applications such as repairing bone defects, drug delivery, and cell delivery.	(368)
PMMA and platelet gel PG	-	In vivo	The modification applied to PMMA increased its regenerative capacity.	(369)
Regenerated silk fibroin with nHAp	-	In vitro	The applied printing scaffold could control its degradation rate by controlling its crystallinity level representing a suitable method for producing dental barrier membranes.	(370)
Lyophilized collagen-polyvinylpyrrolidone sponge	DPSCs	In vivo	Seeding allogeneic DPSCs on this scaffold was suggested as a promising method for treating periodontal bone defects.	(371)
Platelet-rich fibrin	hPDLSCs	In vitro	PRF was successfully used to identify the mechanism and factors involved in the proliferation of alveolar osteoblasts.	(372)
3D-engineered PLA	hGMSCs	In vivo<br>In vitro	The fabricated scaffolds supported bone healing in cortical calvaria bone damage. The used method was suggested as a promising strategy for repairing cranial bone traumas.	(373)
PRP-coated polyethersulfone/PVA	ADSCs	In vitro	The constructed scaffold was ideally osteogenic.	(374)
GelMA macromonomers with HAp and whitlockite NPs	hMSCs	In vitro	The optimal ratio between two main inorganic parts of human bone (HAp and whitlockite) for the maximum osteogenesis by MSCs were identified to be 3-1.	(375)

3D printed PCL and PLGA	hPDLSCs	In vitro	The best ratio between PCL and PLGA for bone regeneration by hPDLSCs was 1-1.	(376)
Electrospun PCL	Primary human MSCs from two tissues	In vitro	Applying oscillatory fluid flow using a straightforward see-saw rocker was suggested as a conveniently available pretreatment for regenerative therapy of small bone defects.	(372)
GO	Murine MSCs	In vitro	GO effects on upregulating cell adhesion and osteogenesis were synergized with a chondrocyte-conditioned medium.	(377)
TiO_2-coated HAp	-	In vivo	TiO_2 coating and UV both increased the bone formation capacity of HAp.	(377)
Poly(vinyl phosphonic acid-co-acrylic acid)	An osteosarcoma cell line and hBMMSCs	In vitro	The polymer concentration was shown to be effective in the osteogenesis activity of cells. The polymer scaffold was suggested for future bone TE for clinical purposes.	(378)
Injectable glass-ceramic (GC) hydrogels	MC3T3-E1 cells	In vivo<br>In vitro	The injectable hydrogel was suggested for dental or orthopedic regenerative procedures.	(379)
TCP particles	-	In vivo	The in situ-hardening TCP used as a bone filler could enhance the mineralization and ideally preserve the hard tissue mandibular third molar and provide a minimally invasive surgical procedure.	(380)
Bioactive glass	SCs derived from dental pulp, dental follicle, and PDL	In vitro	A combination of the scaffold with dental pulp SCs showed the most osteogenesis capacity and was suggested for craniomaxillofacial complex tissue engineering.	(381)
MTA with aminolevulinic acid	hDPCs	In vitro	A photodynamic antibacterial approach was introduced as an effective, cytocompatible, and odontoblastogenic for regenerating a root canal.	(382)
Gelatin and hyaluronic acid copolymer	Mouse embryonic fibroblasts (3T3 cells)	In vitro	The supermacroporous copolymer had ideal properties in terms of mechanical strength, supporting cells, porosity, and injectability.	(383)
Poly3-hydroxybutyrate4-hydroxybutyrate (P34HB)	-	In vivo<br>In vitro	The bioplastic scaffolds had favorable porosity, mechanical and chemical properties, and non-cytotoxicity. They could promote cell adhesion and proliferation and repair calvarial defects.	(62)
3D-printed calcium silicate	hMSCs	In vitro	The structure of these scaffolds was controllable. They showed improved mechanical properties and osteogenic capacity, suggesting them as a potential platform for bone TE.	(384)
Aliphatic polyesters with nHAp and GO nanofillers	-	In vivo<br>In vitro	The porosity of synthesized scaffolds could be precisely controlled. The manufactured biodegradable scaffolds well-supported cell adhesion, proliferation, differentiation, and bone regeneration.	(384)
Gelatin/nHAp microsphere	-	In vitro<br>In vivo	The manufactured scaffold could improve alveolar bone regeneration and be suggested as an efficient alloplastic graft for repairing other bone defects.	(385)
Dypiridamole-loaded bioceramics	-	In vivo	These 3D-printed scaffolds could regenerate natural-like structured bone with good vascularization and mechanical properties.	(236)
PLA, dicalcium phosphate dihydrate, and hydraulic CaSi	Human periapical cyst-derived MSCs	In vitro	The composite seeded with autologous SCs was introduced as a promising approach for regenerative therapy of periapical and alveolar bone defects.	(36)

Polyetherketoneketone	Human synovial fluid MSCs	In vivo<br>In vitro	The 3D-printed scaffold cultivated with SCs could effectively repair critical-sized bone defects.	(385)
Alumina-multiwalled carbon nanotubes	DPSCs	In vitro	The nanoporous scaffold properly supported cell adhesion and proliferation.	(386)
PRF combined with recombinant RGD motif-enriched COLI	-	In vivo	A porous scaffold formed a PRF-FBG complex, which is a good bone graft for clinical applications.	(387)
Nanocrystalline forsterite (FU)	hBMSCs	In vitro	FU showed comparable osteogenic potential with other commercially available materials.	(388)
Graphene	MSCs	In vitro	The mechanosensitive integrin/FAK axis was suggested as the mechanism of graphene osteogenesis.	(389)
Collagen-binding essential fibroblast growth factor	-	In vivo	The GF-embedding scaffold could effectively stimulate bone regeneration.	(390)
Functionalized PLLA	hBMSCs	In vitro	Designed scaffolds could induce osteogenesis in the stem cells.	(391)
Mg⁻CaSi and CH compounds onto Ti⁻6Al⁻4V scaffolds	Human Wharton's Jelly MSCs	In vivo<br>In vitro	The modified scaffold showed promising potential for bone TE purposes.	(392)
PLGA NPs	Human alveolar bone stem cells	In vitro	The PLGA NPs acted as effective nanocarriers with favorable release profiles before polymer degradation.	(393)
Uncalcined HAp/poly-DL-lactide enriched with COL I & III	-	In vivo	The collagen-containing blocks were suggested as promising scaffolds for vertical ridge augmentation.	(394)
PGS	-	In vivo	The more durable PGS membrane was discussed to be a more helpful scaffold.	(395)
3D hexahedron channeled BCP	-	In vivo	The hexahedron channeled blocks were suggested as highly effective bone graft materials.	(396)
3D Gel-CS hybrid hydrogels	BMSCs and ADSCs	In vitro	Using these hybrid hydrogels was suggested as a promising approach for regenerative bone therapy.	(397)
3D-printed PLA	-	In vitro	A topological optimization and manufacturing technology was introduced to optimally design and rapidly print synthetic porous grafts.	(398)
CaP bone graft	-	In vitro	The dense CaP granules were formed with a suitable capacity for regenerating acetabular bone defects.	(399)
Poly(ethylene glycol) diacrylatea-gelatin hydrogel	BMSCs	In vitro	The polymeric hydrogel was suggested as an effective scaffold for treating craniofacial and appendicular skeletal defects and targeted bone TE applications.	(400)
BCP with HAp and β-TCP	-	In vivo	The ceramic composite osteoconductivity conveyed it as a promising material for bone regeneration.	(401)
Ultra-fine titanium mesh membrane	-	In vivo	An adequately stable approach was suggested for GBR and simultaneous implant prostheses.	(402)
GFs-embedded bioCaP	MC3T3-E1 cells	In vitro<br>In vivo	Icariin and BMP-2 were co-administered as GFs and helped bone regeneration.	(398)

Puramatrix™ peptide hydrogel	Human and canine PLSCs	In vivo	The osteogenic scaffold potential increased in the presence of ephrinB2, suggesting it for repairing alveolar bone defects.	(403)
PLGA associated with HAp and β-TCP	-	In vitro<br>In vivo	The studied membranes were biocompatible, and the optimal concentration for absorption and tissue reaction was 500 µm, which was suggested for GBR applications.	(404)
Poly(aspartame) (pAsp)-based hydrogel	PDLCs	In vitro	These cell-gel structures seem to be highly promising for cell-based tissue reconstruction purposes in regenerative medicine.	(405)
pAsp-DNA nanostructures	-	In vitro	The synthetic DNA scaffolds were appropriately functionalized and promoted guided mineralization.	(406)
Retro MTA with β-TCP	-	In vivo	The MTA scaffolds with or without β-TCP covered with a collagen membrane could regenerate periodontal tissues.	(407)
4-hydroxyphenyl 2-(4-hydroxyphenyl)acetate polymer	hMSCs	In vitro	The controllable polymer functionalization helped its applicability for manufacturing various biodegradable scaffolds and medical devices for bone regeneration and fixation with tailored mechanical properties.	(408)
Chitlac-coated thermosets	DPSCs	In vitro	A helpful tool was introduced for assessing cell response to different biomaterials and their engraftment capacity.	(409)
Ion-doped β-TCP and alkali-free bioactive glass	MSCs	In vivo	Optimized doses of therapeutic ions were suggested as a practical approach for using bone grafts for biomedical purposes.	(397)
HAp-β-TCP, powdered PLA, and 3D-printed PLA	-	In vivo	The 3D-printed PLA scaffolds were suggested as a promising material for bone regeneration.	(410)
Reinforced alginate/Gel hydrogels	-	In vitro	Reinforcing by TiO_2 and β-TCP was proposed as a practical approach for fabricating 3D-printed scaffolds with physical properties suitable for bone TE.	(411)
Bilayer PCL	-	In vitro	The nanofibrous PCL bilayer could decrease cell infiltration.	(412)
Modified PMMA bone cement	Fourth passaged MG63	In vitro	The PMMA was modified by dopamine-coated strontium-doped calcium polyphosphate particle bone cement and showed to be a suitable material for bone regeneration.	(413)
L-lysine diisocyanate combined with PEG and PHA	Rat BMSCs	In vitro	The designed composite was recommended for a wide range of biomedical applications.	(413)
PLGA and GO microparticles	Human fetal cartilage rudiment cells	In vitro	GO-PLGA microparticles were cytocompatible and osteoinductive, suggesting their potential for bone TE applications.	(414)
Biphasic HAp/β-TCP and collagen membrane	-	In vivo	The role of socket augmentation, microsurgical instruments, and microsutures under magnification in improving the quality of the regenerated bone and soft tissue was displayed.	(415)
OCP/COL composite	-	In vivo	The composite osteoconductivity depended on the residual healthy bone tissue. Its osteoinductivity promoted angiogenesis and osteogenic cell migration from the host to the bone injury.	(416)
OCP/COL composite	-	In vivo	The composite was suggested for regenerative treatment of skull defects with limited infection risk.	(417)

PLGA-embedded nHAp and nanowhitlockite (nWLKT)	BMSCs	In vivo	Both scaffolds had similar physical, chemical, and cellular properties, but the osteogenic capacity of PLGA/nWLKT was reported to be higher.	(418)
TCP and PLGA	ADSCs	In vivo	The polymeric scaffolds provided a suitable platform for bone regeneration by ADSCs, which is an effective tool for repairing extensive mandibular injuries.	(419)
Fibrous polycarbonate diol, isosorbide-based polyurethane, and hydrophilic nano-GO	C2C12 cells	In vitro	The nanofibrous morphology and high mechanical flexibility of these scaffolds and their myogenic differentiation capacity conveyed them as a promising platform for skeletal muscle TE.	(420)
OCP and bone matrix gelatin (BMG)	-	In vivo	The OCP/BMG was recommended as an optimal matrix for repairing mandibular bone injuries.	(421)
